# Wild passerines as potential carriers and sources of avian influenza viruses in Ukraine

**DOI:** 10.3389/fmicb.2025.1736454

**Published:** 2026-01-20

**Authors:** Nataliia Muzyka, Anastasia Popova, Oleksandr Rula, Polina Yurko, Anzhela Chaplygina, Alexander M. P. Byrne, Abi Lofts, Siamak Zohari, Susanne Koethe, Jeanne Fair, Jen Owen, Nicola Lewis, Anne Pohlmann, Martin Beer, Jonas Waldenström, Denys Muzyka

**Affiliations:** 1 National Scientific Centre, Institute of Experimental and Clinical Veterinary Medicine, Kharkiv, Ukraine; 2 Department of Zoology, H.S. Skovoroda Kharkiv National Pedagogical University, Kharkiv, Ukraine; 3 Worldwide Influenza Centre, WHO Collaborating Centre for Influenza Research and Reference, The Francis Crick Institute, London, United Kingdom; 4 Department of Virology, Immunobiology and Parasitology, National Veterinary Institute, Swedish Veterinary Agency, Uppsala, Sweden; 5 Friedrich-Loeffler-Institute, Greifswald, Germany; 6 Modeling and Observations of Earth Systems, Los Alamos National Laboratory, Los Alamos, NM, United States; 7 Department of Fisheries and Wildlife, Michigan State University, East Lansing, MI, United States; 8 Centre for Ecology and Evolution in Microbial Model Systems, Linnaeus University, Kalmar, Sweden

**Keywords:** avian influenza virus, circulation, H1N1, H3N8, H7N1, intermediate host, Passeriformes, Ukraine

## Abstract

Wild waterfowl and shorebirds are the primary reservoir of influenza A viruses in nature. The role of wild birds from other taxonomic groups remains insufficiently studied or is a subject of debate. This applies in particular to Passeriformes, the most diverse avian order, accounting for approximately 60% of the global bird population, where the role in circulation of influenza A viruses is underexplored. We used serological, virological, and PCR-based methods to survey avian influenza viruses in Passeriformes birds (65 species, 20 families) in Ukraine over a 20-year period, 2004–2025. Antibodies to influenza viruses were detected in serum and egg yolk of seven passerine species, with average seroprevalence 1.24% in sera and 8.94% in yolk samples. Seroprevalence varied across species, ranging from 1.96 to 27.2%. Virological screening resulted in the isolation of two viruses from Fieldfares (*Turdus pilaris*) of the subtypes H1N1 and H7N1. The overall infection rate based on virus isolation was 0.15%, while local infection rate in Fieldfares reached 11.1%. According to PCR results, 41 positive samples were detected, representing 3.61% of all tested birds (ranging from 1.42–9.1%), and by location ranged from 6.25–9.1%. Sequencing and phylogenetic analyses of H1N1 (Fieldfare), H7N1 (Fieldfare), H3N8 (Great Tit *Parus major*) influenza viruses confirmed them as Eurasian lineage low pathogenic avian influenza viruses and with close relatedness to viruses of the same subtypes circulating among wild waterfowl.

## 1 Introduction

Wild birds play a significant role in maintaining the natural circulation of numerous pathogens, including some with zoonotic potential. In fact, it is estimated that approximately 20% of zoonotic pathogens of relevance for human health can be transmitted by wild birds (Woolhouse et al., 2012). One of the most prominent examples is influenza A viruses, where wild birds, primarily waterfowl and shorebirds (i.e., the orders Anseriformes and Charadriiformes), serve as natural reservoirs and a source of large virus subtype and strain diversity (Swayne, 2008). In these hosts, avian influenza viruses (AIV) representing 17 of the 19 known subtypes (H1–H16 and H19) have been detected (Swayne, 2008; Olsen et al., 2006; Bellido-Martín et al., 2026). In addition, influenza A viruses have a high mutation rate and a segmented genome that promotes reassortment, which makes them prone to cross host species barriers (Swayne, 2008). One worrying example is the current near-global epizootic of highly pathogenic avian influenza (HPAI) in wild birds, caused by a H5N1 virus that infects a range of waterfowl and seabirds and with spillover infections in poultry, domestic and wild carnivores, marine mammals, and ruminants (Peacock et al., 2025), underscoring the risk of emergence of a pandemic strain (Bellido-Martín et al., 2026; Lowen et al., 2025) and highlighting the substantial economic impact AIVs have on health and agricultural sectors (Alexakis et al., 2024; Seeger et al., 2021).

Birds other than Anseriformes and Charadriiformes have received significantly less attention regarding their role in influenza virus circulation, including the Passeriformes, despite it being the most diverse avian order (comprising over 6,000 species in approximately 140 families), present in literally every ecosystem, and accounting for around 60% of the global bird population (Williams et al., 2023). Some passerine species are abundant, and several are synanthropic (Shriner and Root, 2020), living in close association with human-modified environments (particularly species from families such as Corvidae, Hirundinidae, Sturnidae, Turdidae, Passeridae, and Fringillidae), which may enhance the risk of pathogen exchange between humans, domestic birds, animals, and birds (Gibb et al., 2020). Moreover, in seasonal environments, a large proportion of Passeriformes species are migratory, potentially capable of transporting pathogens along the migration and contributing to the spread of disease into new geographic areas.

The role of Passeriformes in the ecology of influenza viruses remains a controversial topic (Causey and Edwards, 2008; Peterson et al., 2008; Cumming et al., 2011). Some researchers assert that passerines do not participate in, nor play any role in, the ecology of AIVs (Morishita et al., 1999; Munster et al., 2007), while others argue that they may indeed play a role (Shriner and Root, 2020; Peterson et al., 2008; Chang et al., 2014; Fuller et al., 2010; Kou et al., 2005; Lipkind et al., 1982; Račnik et al., 2008). So far only limited experimental evidence exists, but infection experiments have demonstrated that various low- and highly pathogenic avian influenza virus strains can infect some passerine species, confirming the virus’s ability to replicate in their bodies. Experimental infection studies using two low pathogenic AI strains showed that European starlings *Sturnus vulgaris* could be infected, shed virus, and seroconvert (Qin et al., 2011). Experiments with Red-billed queleas *Quelea quelea* and blackcaps *Sylvia atricapilla* shows virus reproduction, virus shedding after oculo-oronasally inoculation of HPAI H5N1 (Breithaupt et al., 2011) as well as American robins *Turdus migratorius* after experimental infection of HPAI H5N2 and H5N8 (Root et al., 2018). Given their abundance in farm environments, passerines have been hypothesized as potential “bridge species” allowing virus transmission among different bird groups (Caron et al., 2014), including domestic poultry, wild waterfowl, chickens, and turkeys (Ayala et al., 2020). Literature data indicates that approximately 0.1% of passerines had detectable antibodies to influenza viruses (Slusher et al., 2014), although other reports cite much higher seropositivity levels ranging from 6 to 76% (Hadipour et al., 2011). Virus isolation studies have shown an average prevalence of 0.5% among passerines (Slusher et al., 2014), while PCR-based detection rates ranged from 0.1–1% (Fuller et al., 2010; Slusher et al., 2014; Hesterberg et al., 2009; Cappelle et al., 2012; Kou et al., 2009; Williams et al., 2012) to 2.3–17.5% in other studies (Peterson et al., 2008; Cumming et al., 2011; Qin et al., 2011; Borovská et al., 2011; Gronesova et al., 2008; Gronesova et al., 2008; Thinh et al., 2012), and in some cases as high as 50% (Fuller et al., 2010). Most positive results of isolation (77%) were associated with sampling of birds in an unnatural setting (recognized poultry outbreaks of LPAI or HPAI, live-bird markets, or when samples only included peridomestic species). A minority of the AIV isolations (23%) were from terrestrial birds in natural settings. For PCR surveillance most of positive samples were obtained from a natural setting but some of them were associated with outbreaks (Slusher et al., 2014). New wave of broad-scale surveillance of wild birds, including Passeriformes, has intensified during the global panzootic of H5 highly pathogenic avian influenza (HPAI) since 2020. During this period, between 1.1 and 6.5% of Passeriformes tested positive for influenza virus by PCR, and in most cases these detections involved H5 HPAI viruses (European Food Safety Authority, European Centre for Disease Prevention and Control, 2022; Günther et al., 2023).

Research on the ecology of influenza viruses in wild birds in Ukraine began in the early 2000s and has primarily focused on wild waterfowl and shorebirds (Muzyka et al., 2012; Muzyka et al., 2016; Muzyka et al., 2016; Muzyka et al., 2019). Circulation of this pathogen among other ecological bird groups has received little attention. Currently, the order Passeriformes in Ukraine includes 165 species that are widely distributed and inhabit a variety of ecosystems, including urbanized and synanthropic environments. Given the current epizootic situation, the widespread circulation of HPAI viruses among wild birds, poultry, and mammals, and the potential threat to human health, interest has grown in identifying new sources and reservoirs that may contribute to the natural circulation of pathogens (Williams et al., 2023). In this context, we have renewed our focus on wild passerine birds. This article summarizes the results of 20 years of research on Passeriformes in Ukraine.

## 2 Materials and methods

### 2.1 Study area and field samples

**Figure 1:**
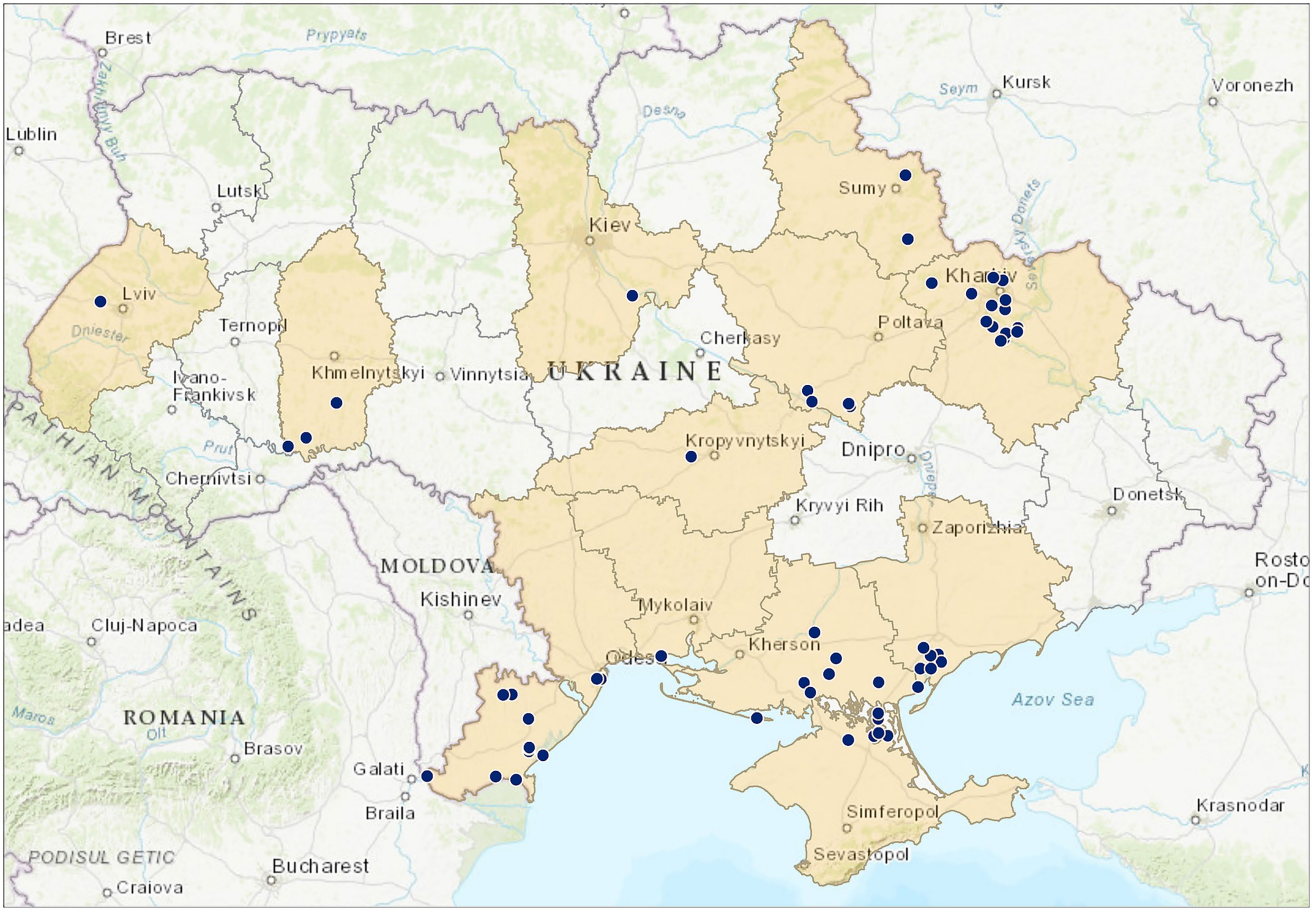
Main field sampling location of *Passeriformes* in Ukraine in 2004–2025.

Wild passerines have been sampled as part of active surveillance efforts in Ukraine since 2004, but with variable efforts between years. Sampling was carried out in different geographical regions in 12 oblasts (Kharkiv, Sumy, Poltava, Kyiv, Zaporizhzhia, Kherson, Odesa, Lviv, Mykolaiv, Kirovohrad, Khmelnytskyi Oblasts, AR Crimea; Figure 1), representing different ecological habitats (natural, urban, synanthropic) and birds with different ecological status (e.g., breeding species, migratory species, wintering species, nomadic species, resident species) in 2004–2025. During the periods 2004–2007 and 2023–2025, a total of 483 blood serum samples and 302 egg yolk samples were collected from Passeriformes (38 species, 14 families). For virological investigations conducted between 2010 and 2021, 1,280 biological samples (fecal and cloacal swabs) were collected (38 species, 13 families). In the period 2023–2025, 1,134 biological samples were collected for PCR testing from birds representing 50 species and 19 families. The list of species and samples are provided in Table 1 and in the Supplementary Table S1.

**Table 1:** Overview of bird’s samples of order Passeriformes analyzed in this study.

Family	Species	Samples, *n*			
		Virology	PCR	Serology	Eggs Yolk
Aegithalidae	1	1	16	–	–
Alaudidae	2	61	–	–	–
Bombycillidae	1	–	10	–	–
Certhiidae	1	–	1	–	–
Corvidae	5	529	11	19	26
Emberizidae	3	26	11	16	1
Fringillidae	4	43	204	111	12
Hirundinidae	3	267	33	28	16
Laniidae	2	–	10	5	5
Motacillidae	3	11	5	2	–
Muscicapidae	12	48	233	124	100
Oriolidae	1	-	4	4	–
Panuridae	1	8	2	2	-
Paridae	3	11	252	55	30
Passeridae	3	59	139	69	109
Regulidae	1	–	12	–	–
Sittidae	1	–	4	1	–
Sturnidae	1	187	48	5	–
Sylviidae	16	29	136	42	3
Troglodytidae	1	–	3	–	–
Total: 20	65	1,280	1,134	483	302

### 2.2 Serological studies

Antibodies to avian influenza virus were detected in egg yolk and blood serum. Blood samples and corresponding serum samples were obtained from captured wild birds using standard techniques (Spackman, 2008; Delany et al., 2007). Obtaining and preparing egg yolks from wild birds was carried out as previously described (Muzyka et al., 2016), with a slight modification consisting of 10–15% chloroform added to the egg yolk solution and mixed for 1–2 min for removing lipids. Serum and egg yolk were stored at −20 °C prior to testing. Antibodies to the influenza virus were detected in a hemagglutination inhibition (HI) test using inactivated hemagglutinating antigens of subtypes H1-H16 following established methods (Spackman, 2008; Spackman, 2014; American Association of Avian Pathologists, 2008). In addition, two commercial ELISA kits were also used for serology testing (IDEXX AI MultiS-Screen Ab Test, The Netherlands; ID Screen® Influenza A Antibody Competition Multi-species, ID Vet, France).

### 2.3 Virology studies

Virus isolation from the samples was conducted in accordance with the WOAH procedure (Swayne, 2008; Spackman, 2014; American Association of Avian Pathologists, 2008). Swabs and fecal samples were inoculated into the allantoic cavity of 9–10 day-old specific pathogen free embryonated hens’ eggs (Valo BioMedia GmbH, Germany). Every sample was passaged three times. The presence of hemagglutinating viruses in allantoic fluid was determined with a 1% suspension of chicken red blood cells. The hemagglutinin (HA) virus subtype was determined by HI tests using the following antisera: H1N1, H2N3, H3N8, H4N6, H4N8, H5N1, H5N2, H5N3, H5N8, H6N2, H6N8, H7N1, H7N2, H7N3, H8N4, H9N2, H9N7, H10N1, H10N7, H10N9, H11N6, H11N9, H12N5, H13N6, H14N5, H14N6, H15N9, H16N3, APMV-1, APMV-2, APMV -3, APMV-4, APMV-6, APMV-7, APMV-8, APMV-9 (provided by Veterinary Laboratories Agency, Weybridge, UK and Instituto Zooprofilattio Sperimentale delle Venezie, Padova, Italy).

### 2.4 PCR

RNA from cloacal swabs and fecal samples was extracted using the Quick-DNA/RNA Viral Kit (ZYMO RESEARCH, United States) according to the manufacturer’s instructions. Detection of the virus was performed by RT-qPCR targeting the M gene according to previously described methods (Nagy et al., 2021). All influenza A virus-positive samples were investigated using HA and NA subtype specific RT-PCRs (Hassan et al., 2022; Slomka et al., 2007; Van Borm et al., 2010). The reaction mixture was prepared using the commercial AgPath-ID™ One-Step RT-PCR Reagents kit (Applied Biosystems, United States) and RT-qPCR carried out using a QuantStudio™ 5 Real-Time PCR System (Applied Biosystems, United States).

### 2.5 Sequencing and phylogenetic analysis

Three viruses from Passeriformes were chosen for whole genome sequencing. The H7N1 and H1N1 viruses isolated from Fieldfare (*Turdus pilaris*) and H3N8 virus detected in PCR in Great Tit *Parus major* were sequenced at the Friedrich-Loeffler-Institute (Riems, Germany) and at the Swedish Veterinary Agency (Uppsala, Sweden). RNA was amplified with influenza-specific primers using Invitrogen Superscript III One-Step RT-PCR with Platinum Taq (ThermoFisher Scientific). After amplification, samples were purified with AMPure XP Magnetic Beads (Beckman Coulter. Fullerton. United States). Quantification was conducted with the NanoDrop™ 1,000 Spectrophotometer (ThermoFisher Scientific). For MinION library preparation the Rapid Barcoding Kit (RBK-004. ONT) was applied following the manufacturer’s instructions. Following the library preparation, the pooled samples were loaded onto a FLO-MIN107 R9.4.1 flow cell following the manufacturer’s instructions (ONT) (King et al., 2020). For IonTorrent Sequencing the RT-PCR amplicons were sequenced as previously described (Pohlmann et al., 2017; Pohlmann et al., 2018). Consensus sequences were generated using an iterative mapping approach using Bowtie2 (v. 2.3.0) in the Geneious software suite (Biomatters, Auckland, New Zealand). Geneious software suite was also used for quality check and automatic annotation of the sequences. One virus (H3N8) detected by PCR in Grate Tit was partial genome sequenced at the Francis Crick Institute (London, United Kingdom). Influenza genome segments were amplified with Influenza-specific primers using OneStep Ahead RT-PCR kit (Qiagen). Sequencing libraries were generated using the QIAseq FX DNA Library kit (Qiagen) and sequenced on an Illumina MiSeq. Raw sequence data were assembled using an in-house pipline as described previously (Byrne Alexander et al., 2023).

The sequences of the final data set were then aligned for each gene using MAFFT and manually trimmed to the open reading frame using AliView. Phylogenetic trees were then inferred using the maximum likelihood approach in IQ-Tree with ModelFinder to infer the appropriate phylogenetic model and 1,000 ultrafast bootstraps (Byrne Alexander et al., 2023).

GenBank accession numbers for the viruses sequenced are: PX091979, PX726955 (GenBank), EPI4242121 (GISAID).

## 3 Results

### 3.1 Serological study

Influenza virus antibodies were detected by ELISA or HI in 6 of 483 serum samples collected 2004–2025. These samples came from 6 species: Hooded Crow (*Corvus cornix*), Common Blackbird (*Turdus merula*), Eurasian Blackcap (*Sylvia atricapilla*), Eurasian Jay (*Garrulus glandarius*), Common Reed Bunting (*Emberiza schoeniclus*), and Song Thrush (*Turdus philomelos*) in Poltava, Kharkiv, Odesa, and Khmelnytskyi oblasts. Additionally, antibodies were detected in 27 (out of 302) egg yolk samples from seven species: Common Blackbird, Collared Flycatcher (*Ficedula albicollis*), Eurasian Jay (*Garrulus glandarius*), Song Thrush, Bluethroat (*Luscinia svecica*), Great Tit (*Parus major*), and House Sparrow (*Passer domesticus*) (Table 2). These results integrate previously published data with newly obtained findings (Popova and Muzyka, 2025). Positive samples came from various Ukrainian regions (Eastern, Central, Western, and Southern) during both the migration and breeding periods. The overall average seroprevalence of avian influenza among Passeriformes in Ukraine during 2004–2025 was 1.2% in serum samples and 8.9% in egg yolks. Seropositivity varied among species, ranging from 1.9 to 20.6%, depending on species. (Table 2). The full data is presented in Supplementary Table S2.

**Table 2:** Detection AIV antibody in passerines in Ukraine in 2004–2025 (positive only).

Bird species	Oblast	Location	Year	Samples, total	Test type	Result		Prevalence	
						Pos	Neg	In location, %	In species, %
Serum									
Corvidae									
Hooded Crow *Corvus cornix*	Zaporizhzhia Oblast	Poultry Farm	2004	4	Н1-Н14	1	3	25.0 (95% CI: 0.0–67,4%)	25 (95% CI: 0.0–67,4%)
Jay *Garrulus glandarius*	Poltava Oblast	RLP Nizhnevorsklyanskiy	2023	1	ELISA	0	1	0	12.5 (95% CI: 0.0–35,4%)
			2024	2	ELISA	0	2	0	
			2025	2	ELISA	1	1	50.0*	
	Kyiv Oblast	Balyko-Schynsenka	2023	2	ELISA	0	2	0	
	Kharkiv Oblast	Pershotravneve	2024	1	ELISA	0	1	0	
Emberizidae									
Reed Bunting *Emberiza schoeniclus*	Odesa Oblast		2024	10	ELISA	1	9	10.0 (95% CI: 0.0–28,5%)	10 (95% CI: 0.0–28,5%)
Muscicapidae									
Blackbird *Turdus merula*	Kharkiv Oblast	Gaidary	2023	7	ELISA	0	7	0	1.96 (95% CI: 0.0–5,7%)
		Pershotravneve	2024	9	ELISA	0	9	0	
			2025	1	ELISA	0	1	0	
	Khmelnytskyi Oblast	Maliivtsi	2023	1	ELISA	0	1	0	
		NNP «Podilski Tovtry»	2024	1	ELISA	0	1	0	
	Poltava Oblast	RLP Nizhnevorsklyanskiy	2023	10	ELISA	1	9	10.0 (95% CI: 0.0–28,5%)	
			2024	14	ELISA	0	14	0	
			2025	6	ELISA	0	6	0	
	Kyiv Oblast	Balyko-Shchuchinka	2023	2	ELISA	0	2	0	
Song Thrush *Turdus philomelos*	Kharkiv Oblast	Gaidary	2023	7	ELISA	0	7	0	2.04 (95% CI: 0.0–5,9%)
		Pershotravneve	2024	5	ELISA	1	4	20.0 (95% CI: 0.0–55,6%)	
			2025	1	ELISA	0	1	0	
	Khmelnytskyi Oblast	NNP «Podilski Tovtry»	2024	1	ELISA	0	1	0	
	Poltava Oblast	RLP Nizhnevorsklyanskiy	2023	10	ELISA	0	10	0	
			2024	21	ELISA	0	21	0	
			2025	3	ELISA	0	3	0	
	Odesa Oblast	NPP Tuzlivski Lymany	2024	1	ELISA	0	1	0	
Sylviidae									
Blackcap *Sylvia atricapilla*	Odesa Oblast	Trapivka	2024	1	ELISA	0	1	0	12,5 (95% CI: 0.0–34,4%)
		Lyman	2024	1	ELISA	0	1	0	
		Trapivka-2	2024	3	ELISA	0	3	0	
	Poltava Oblast	RLP Nizhnevorsklyanskiy	2024	2	ELISA	0	2	0	
	Khmelnytskyi Oblast	NNP «Podilski Tovtry»	2024	1	ELISA	1	0	100*	
Eggs yolk									
Corvidae									
Jay *Garrulus glandarius*	Kharkiv Oblast	Kharkiv (Hidropark)	2006	1	Н1, H8	1	0	100*	50*
	Kirovograd Oblast	Ukrainka	2025	1	ELISA	0	1	0	
Muscicapidae									
Blackbird *Turdus merula*	Kharkiv Oblast	Haidary	2006	4	Н1–Н14	0	4	0	11.1 (95% CI: 0.0–31,6%)
		Hineievka	2006	2	Н1	1	1	50,0*	
	Khmelnytskyi Oblast	Maliivtsi	2023	1	ELISA	0	1	0	
	Kharkiv Oblast	Gaidary	2023	1	ELISA	0	1	0	
	Kirovograd Oblast	Ukrainka	2025	1	ELISA	0	1	0	
Collared Flycatcher *Ficedula albicollis*	Sumy Oblast	Vakalivshchyna,	2004	3	Н1–Н14	0	3	0	5.0 (95% CI: 0.0–11,7%)
		NNP «Hetmanskyi»	2006	9	Н1	2	7	22,2 (95% CI: 0.0–49,3%)	
	Kharkiv Oblast	Haidary	2006	11	Н1–Н14	0	11	0	
		RLP «Feldman ecopark»	2006	9	Н1–Н14	0	9	0	
		NNP «Homilshanski lisy»	2006	6	Н1–Н14	0	6	0	
		Hineievka	2006	2	Н1–Н14	0	2	0	
Song Thrush *Turdus philomelos*	Poltava Oblast	RLP Nizhnevorsklyanskiy	2023	1	ELISA	0	1	0	20.6 (95% CI: 5,9–35,4%)
	Khmelnytskyi Oblast	Maliivtsi	2023	1	ELISA	0	1	0	
	Sumy Oblast	NNP «Hetmanskyi»	2006	2	Н1	2	0	100,0*	
		Vakalivshchyna	2006	1	Н1–Н14	0	1	0	
	Kharkiv Oblast	Haidary	2006	11	Н1–Н14	0	11	0	
		Kharkiv (Hydropark)	2006	1	Н1–Н14	0	1	0	
		NNP «Homilshanski lisy»	2006	4	Н1	2	2	50,0 (95% CI: 1.0–99,0%)	
					Н2	1	3	25,0 (95% CI: 0.0–67,4%)	
					Н8	1	3	25,0 (95% CI: 0.0–67,4%)	
			2007	1	Н1–Н14	0	1	0	
Bluethroat *Luscinia svecica*	Kharkiv Oblast	Haidary	2007	1	Н5, Н14	1	0	100,0*	100*
Paridae									
Great Tit *Parus major*	Poltava Oblast	Horishni Plavni	2023	1	ELISA	0	1	–	7.4 (95% CI: 0.0–17,2%)
	Kharkiv Oblast	RLP «Feldman ecopark»	2006	1	Н1–Н14	0	1	0	
		NNP «Homilshanski lisy»	2006	14	Н1–Н14	0	14	0	
	Sumy Oblast	NNP «Hetmanskyi»	2006	7	Н1–Н14	0	7	0	
		Vakalivshchyna	2006	2	Н1	1	1	50,0*	
					Н2, H8	1	1	50,0*	
Passeridae									
House Sparrow *Passer domesticus*	Kharkiv Oblast	Poultry farm №1	2004	40	Н1	6	34	15,0 (95% CI: 3.9–26,6%)	8,69 (95% CI: 2.0–15,3%)
		Poultry farm №3	2004	29	Н1–Н14	0	29	0	

*CI has not counted due small numbers of samples.

Antibodies to influenza A virus were detected in the blood serum of a Hooded Crow (subtype H1, HI, 2004), sampled near a poultry facility in Zaporizhzhia Oblast (urbanized landscape type). In all other cases, antibodies were detected in birds from natural environments: Eurasian Jay (ELISA, Poltava Oblast, 2025), Common Reed Bunting (ELISA, Odesa Oblast, 2024), Common Blackbird (ELISA, Poltava Oblast, 2023), Song Thrush (ELISA, Kharkiv Oblast, 2024), and Eurasian Blackcap (ELISA, Khmelnytskyi Oblast, 2024). At the same time, serological testing of egg yolks revealed a greater number of positive species with antibodies to various AIV subtypes: House Sparrow (Kharkiv Oblast, HI, H1, H8, 2004), Collared Flycatcher (Sumy Oblast, HI, H1, 2006), Song Thrush (Kharkiv and Sumy oblasts, HI, H1, H2, H8, 2006), Great Tit (Sumy Oblast, HI, H1, H2, H8, 2006), Eurasian Jay (Kharkiv Oblast, HI, H1, H8), Common Blackbird (Kharkiv Oblast, HI, H1, 2006), and Bluethroat (Kharkiv Oblast, HI, H5, H14, 2007). Seroprevalence among species ranged from 12.5 to 100%, depending on the species.

### 3.2 Virological study

Between 2010 and 2021, a total of 1,280 fecal samples and cloacal swabs were collected from Passeriformes representing 38 species from 13 families across seven oblasts of Ukraine. Three of the samples yielded hemagglutinating viruses after egg passage, one from 2010 and two from 2021, all from southern Ukraine (AR Crimea and Zaporizhzhia Oblast, respectively). The first virus, obtained from a Common Starling (*Sturnus vulgaris*) was identified as avian paramyxovirus serotype 4 (APMV-4/starling/Medvedkovo/Ukraine/5–24-12/2010) (Reeves et al., 2016). The other two isolates were obtained from Fieldfares and classified as influenza A viruses of the subtypes H1N1 (A/Fieldfare/Bogatyr-Ukraine/M218914/86–90/24–02/2021) and H7N1 (A/Fieldfare/Bogatyr-Ukraine/M2110904/15–18/24–02/2021). These viruses were isolated during the wintering period in southern Ukraine (Zaporizhzhia Oblast) and the local infection rate among sampled Fieldfares was 11.1%. The overall influenza virus prevalence among Passeriformes during the 2010–2021 was 0.15%.

### 3.3 PCR

In total, samples from 1,134 individual passerine birds collected in seven oblasts of Ukraine were analyzed. AIV RNA was detected in 41 samples from 16 species representing six bird families: Fringillidae (Eurasian Chaffinch *Fringilla coelebs*, European Goldfinch *Carduelis carduelis*, European Greenfinch *Chloris chloris*), Muscicapidae (Blackbird, Song Thrush, Collared Flycatcher, European Robin *Erithacus rubecula*,), Panuridae (Bearded Reedling *Panurus biarmicus*), Paridae (Great Tit), Passeridae (House Sparrow), Sylviidae (Eurasian Blackcap *Sylvia atricapilla*, Common Chiffchaff *Phylloscopus collybita*, Lesser Whitethroat *Curruca curruca*, Common Reed Warbler *Acrocephalus scirpaceus*, Sedge Warbler *Acrocephalus schoenobaenus*, Savi’s Warbler *Locustella luscinioides*). Positive detections were reported in four oblasts: Kharkiv, Odesa, Lviv, and Poltava. The overall PCR detection rate among Passeriformes in Ukraine during 2023–2025 was 3.6%, with species-specific infection rates between 1.4–12.0%. Detection rates by location ranged from 6.3–25.0%, with limited cases reaching as high as 50–100% in specific sampling events (Table 3; Supplementary Table S3). None of the M-gene positive samples were subsequently positive in H5 and H7 PCRs.

**Table 3:** Results of PCR detection of AIV in passerines in Ukraine in 2022–2025 (positive results only).

Bird species	Oblast	Location	Year	Samples, total	PCR		Prevalence	
					Pos	Neg	In location, %	In species, total, %
Fringillidae								
Chaffinch *Fringilla coelebs*	Kharkiv Oblast	Gaidary	2023	22	0	22	0	5,26 (95% CI: 0.0–11,0%)
		Pershotravneve	2024	5	1	4	20 (95% CI: 0.0–55,0%)	
	Khmelnytskyi Oblast	Maliivtsi	2023	5	0	5	0	
	Poltava Oblast	RLP Nyzhnovorsklianskyi	2024	17	0	17	0	
			2025	8	2	6	25 (95% CI: 0.0–55,0%)	
Goldfinch *Carduelis carduelis*	Kharkiv Oblast	Gaidary	2023	6	1	5	16,6 (95% CI: 0.0–46,4%)	6,6 (95% CI: 0.0–19,2%)
	Poltava Oblast	RLP Nyzhnovorsklianskyi	2023	6	0	6	0	
			2024	2	0	2	0	
			2025	1	0	1	0	
Greenfinch *Chloris chloris*	Kharkiv Oblast	Gaidary	2023	8	0	8	0	4,8 (95% CI: 0.6–8,9%)
	Khmelnytskyi Oblast	Maliivtsi	2023	2	0	2	0	
	Poltava Oblast	RLP Nyzhnovorsklianskyi	2023	43	2	41	4,6 (95% CI: 0.0–10,9%)	
			2024	21	0	21	0	
			2025	30	3	27	10	
Muscicapidae								
Blackbird *Turdus merula*	Kharkiv Oblast	Gaidary	2023	11	2	9	18,1 (95% CI: 0.0–40,7%)	6,15 (95% CI: 0.3–12,0%)
		Pershotravneve	2024	9	1	8	11,1 (95% CI: 0.0–31,6%)	
	Khmelnytskyi Oblast	Maliivtsi	2023	6	0	6	0	
		NPP «Podilski Tovtry»	2024	1	0	1	0	
	Poltava Oblast	RLP Nyzhnovorsklianskyi	2023	11	1	10	9	
			2024	12	0	12	0	
			2025	10	0	10	0	
	Kyiv Oblast	Hlyboki Balyky	2023	2	0	2	0	
	Kirovohrad Oblast	Ukrainka	2025	3	0	3	0	
Collared Flycatcher *Ficedula albicollis*	Khmelnytskyi Oblast	Maliivtsi	2023	4	0	4	0	9,09 (95% CI: 0.0–26,0%)
	Odesa Oblast	Lyman	2024	5	0	5	0	
		Trapivka-2	2024	2	1	1	50 (95% CI: 0.0–100,%)	
Robin *Erithacus rubecula*	Kharkiv Oblast	Gaidary	2023	10	2	8	20 (95% CI: 0.0–44,7%)	4,05 (95% CI: 0.0–8,5%)
		Pershotravneve	2024	4	0	4	0	
	Poltava Oblast	RLP Nyzhnovorsklianskyi	2023	1	0	1	0	
			2024	24	1	23	4,1 (95% CI: 0.0–12,1%)	
			2025	17	0	17	0	
	Kyiv Oblast	Hlyboki Balyky	2023	9	0	9	0	
	Khmelnytskyi Oblast	NPP «Podilski Tovtry»	2024	6	0	6	0	
	Lviv Oblast	NPP Yavorivskyi	2024	3	0	3	0	
Song Thrush *Turdus philomelos*	Kharkiv Oblast	Gaidary	2023	17	0	17	0	1,5 (95% CI: 0.0–4,4%)
		Pershotravneve	2024	7	0	7	0	
	Poltava Oblast	RLP Nyzhnovorsklianskyi	2023	13	0	13	0	
			2024	21	0	21	0	
			2025	7	1	6	14,2 (95% CI: 0.0–40,2%)	
	Khmelnytskyi Oblast	NPP «Podilski Tovtry»	2024	1	0	1	0	
Panuridae								
Bearded Tit *Panurus biarmicus*	Odesa Oblast	NPP Tuzlivski Lymany	2024	2	2	0	100*	100
Paridae								
Great Tit *Parus major*	Kharkiv Oblast	Dergachi	2022	2	0	2	0	5,06 (95% CI: 2.1–7,9%)
		Manchenki	2022	55	0	55	0	
		Pershotravneve	2023	22	5	17	22,7 (95% CI: 5.2–40,2%)	
			2024	14	0	14	0	
		Gaidary	2023	29	2	27	6,8 (95% CI: 0.0–16,1%)	
	Poltava Oblast	RLP Nyzhnovorsklianskyi	2023	13	0	13	0	
			2024	16	1	15	6,25 (95% CI: 0.0–18,1%)	
			2025	23	1	22	4,3 (95% CI: 0.0–12,6%)	
	Kyiv Oblast	Hlyboki Balyky	2023	15	0	15	0	
	Lviv Oblast	NPP Yavorivskyi	2024	17	1	16	5,8 (95% CI: 0.0–17,0%)	
	Odesa Oblast	Liman	2024	2	0	2	0	
		Trapivka	2024	1	0	1	0	
		Trapivka-2	2024	5	1	4	20 (95% CI: 0.0–55,0%)	
	Khmelnytskyi Oblast	NPP «Podilski Tovtry»	2024	2	0	2	0	
	Kirovohrad Oblast	Ukrainka	2025	1	0	1	0	
Passeridae								
House Sparrow *Passer domesticus*	Kharkiv Oblast	Dergachi	2022	33	0	33	0	1,42 (95% CI: 0.0–4,2%)
		Manchenki	2022	29	0	29	0	
		Pershotravneve	2023	8	1	7	12,5 (95% CI: 0.0–35,4%)	
Sylviidae								
Blackcap *Sylvia atricapilla*	Khmelnytskyi Oblast	Maliivtsi	2023	2	0	2	0	3,33 (95% CI: 0.0–9,7%)
		NPP «Podilski Tovtry»	2024	4	0	4	0	
	Poltava Oblast	RLP Nyzhnovorsklianskyi	2023	6	0	6	0	
			2024	4	0	4	0	
			2025	8	1	7	12,5 (95% CI: 0.0–35,4%)	
	Odesa Oblast	Lyman	2024	1	0	1	0	
		Trapivka-2	2024	3	0	3	0	
	Kirovohrad Oblast	Ukrainka	2025	2	0	2	0	
Chiffchaff *Phylloscopus collybita*	Poltava Oblast	RLP Nyzhnovorsklianskyi	2023	2	0	2	0	5,26 (95% CI: 0.0–15,3%)
			2024	4	0	4	0	
			2025	10	0	10	0	
	Kharkiv Oblast	Pershotravneve	2024	1	0	1	0	
	Odesa Oblast	NPP Tuzlivski Lymany	2024	1	1	0	100*	
	Kirovohrad Oblast	Ukrainka	2025	1	0	1	0	
Lesser Whitethroat *Sylvia curruca*	Odesa Oblast	Liman	2024	9	1	8	11,1 (95% CI: 0.0–31,6%)	12,0 (95% CI: 0.0–24,7%)
		Trapivka	2024	11	0	11	0	
		Trapivka-2	2024	4	1	3	25 (95% CI: 0.0–67,4%)	
	Poltava Oblast	RLP Nyzhnovorsklianskyi	2025	1	1	0	100*	
Reed Warbler *Acrocephalus scirpaceus*	Odesa Oblast	Liman	2024	16	1	15	6,25 (95% CI: 0.0–18,1%)	5,26 (95% CI: 0.0–15,3%)
		Trapivka	2024	2	0	2	0	
		Trapivka-2	2024	1	0	1	0	
Savi’s Warbler *Locustella luscinioides*	Odesa Oblast	Liman	2024	1	1	0	100*	100*
Sedge Warbler *Acrocephalus schoenobaenus*	Odesa Oblast	NPP Tuzlivski Lymany	2024	2	2	0	100*	100*

*CI has not counted due small numbers of samples.

### 3.4 Sequencing and phylogenetic analysis

**Figure 2:**
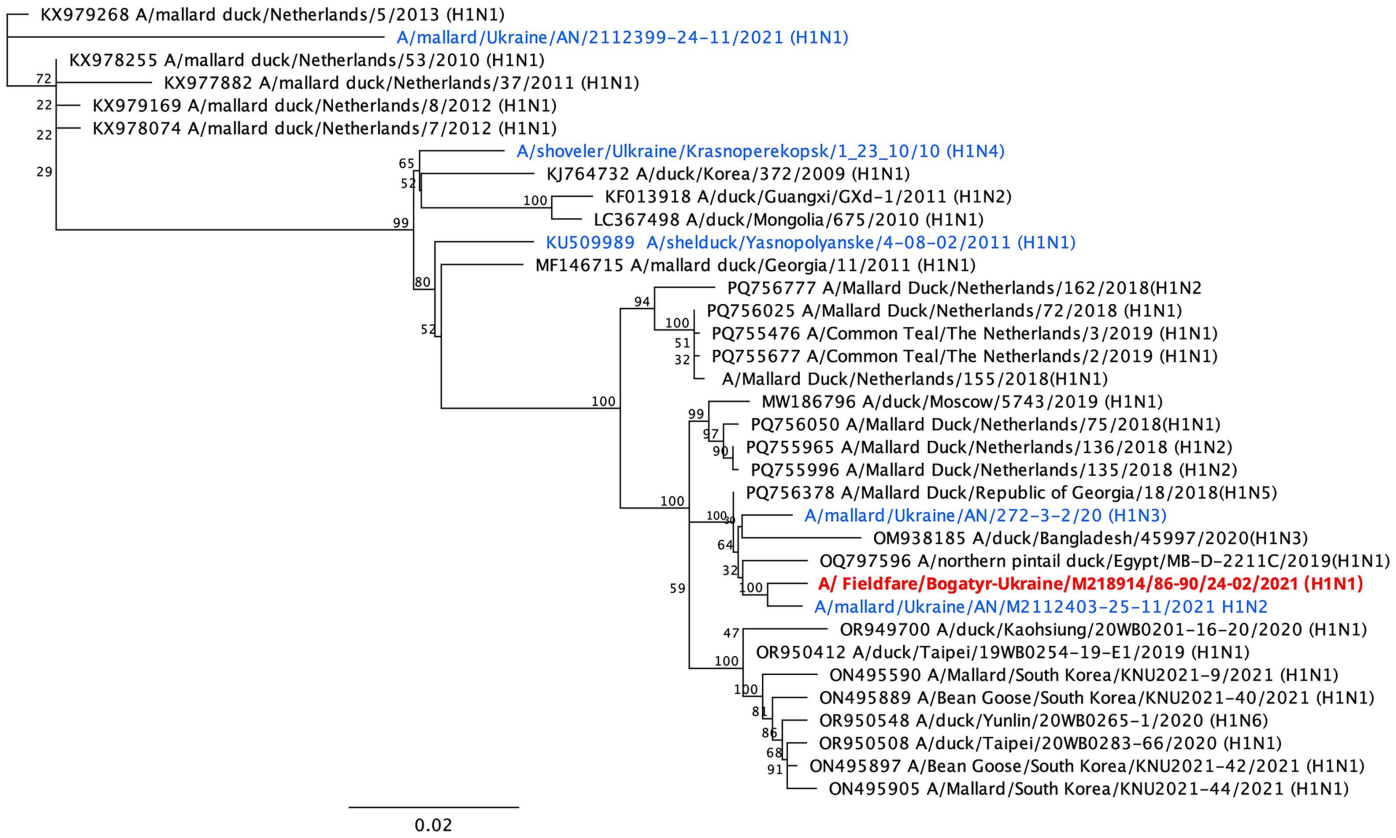
Phylogenetic tree constructed based on the results of sequencing the complete HA gene of LPAIV H1N1 subtype isolated in Fieldfare in Ukraine (red color). Samples in blue are other viruses the same subtype isolated from wild birds in Ukraine.

For sequencing, the 15 PCR-positive samples with low Ct values (<35) were selected, along with the two influenza virus isolates obtained from Fieldfares. According to the study results, only three samples were sufficient for future analyses. Next-generation sequencing yielded full-genome sequences for the two Fieldfare isolates (H1N1 and H7N1), as well as a partial genome sequence (H3N8) from a PCR-positive sample from a Great Tit. The HA gene sequences of these viruses were used for phylogenetic analysis. Together with sequences of influenza A viruses of subtypes H1, H3, and H7 isolated from wild waterfowl in Ukraine between 2010 and 2021. Phylogenetic analysis revealed that all viruses isolated/ detected in Passeriformes clustered with AIVs of the same subtypes that were previously identified in wild waterfowl, particularly within the order Anseriformes, genus *Anser*. The Fieldfare H1N1 virus belonged (cleavage site PSIQSR↓GLF) to the LPAI Eurasian lineage of H1 viruses circulating between 2019 and 2021 (Figure 2), and was closely related to an H1N2 virus obtained from Mallard *Anas platyrhynchos* in the same region and time period, and it clustered with an H1N3 virus previously isolated from wild waterfowl in Ukraine, as well as with H1 viruses from Egypt, Bangladesh, and Georgia. In contrast, earlier H1 viruses isolated from wild waterfowl in Ukraine during 2010–2011 were positioned in distinct phylogenetic clusters, indicating temporal divergence within the Ukrainian H1 viruses.

**Figure 3:**
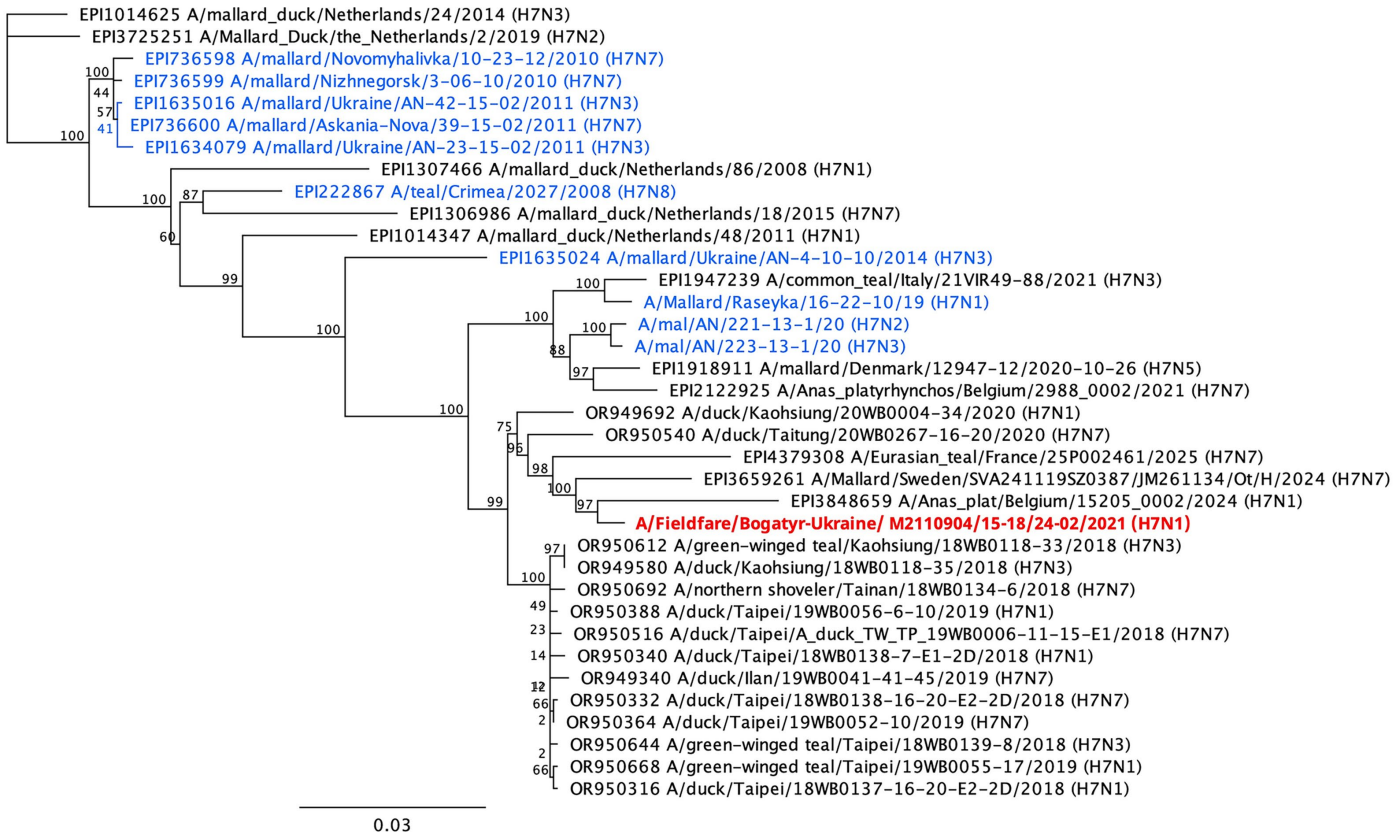
Phylogenetic tree constructed based on the results of sequencing the complete HA gene of LPAIV H7N1 subtype isolated in fieldfare in Ukraine (red color). Samples in blue are other viruses the same subtype isolated from wild birds in Ukraine.

Structure of the HA cleavage site (EIPKGR↓GLF) of the Fieldfare H7N1 isolate confirmed it as a LPAI virus. Phylogenetic analysis indicated that the virus belongs to the Eurasian lineage of H7 AIV circulating among wild waterfowl during 2018–2024. Notably, this isolate clustered with H7 viruses that circulated in Western Europe during 2024–2025 and showed high similarity to an H7N1 virus isolated from mallard in Belgium. In contrast, all other Ukrainian H7 influenza viruses isolated from wild waterfowl between 2008 and 2020 formed separate clusters, though still within the broader European H7 lineage (Figure 3).

**Figure 4:**
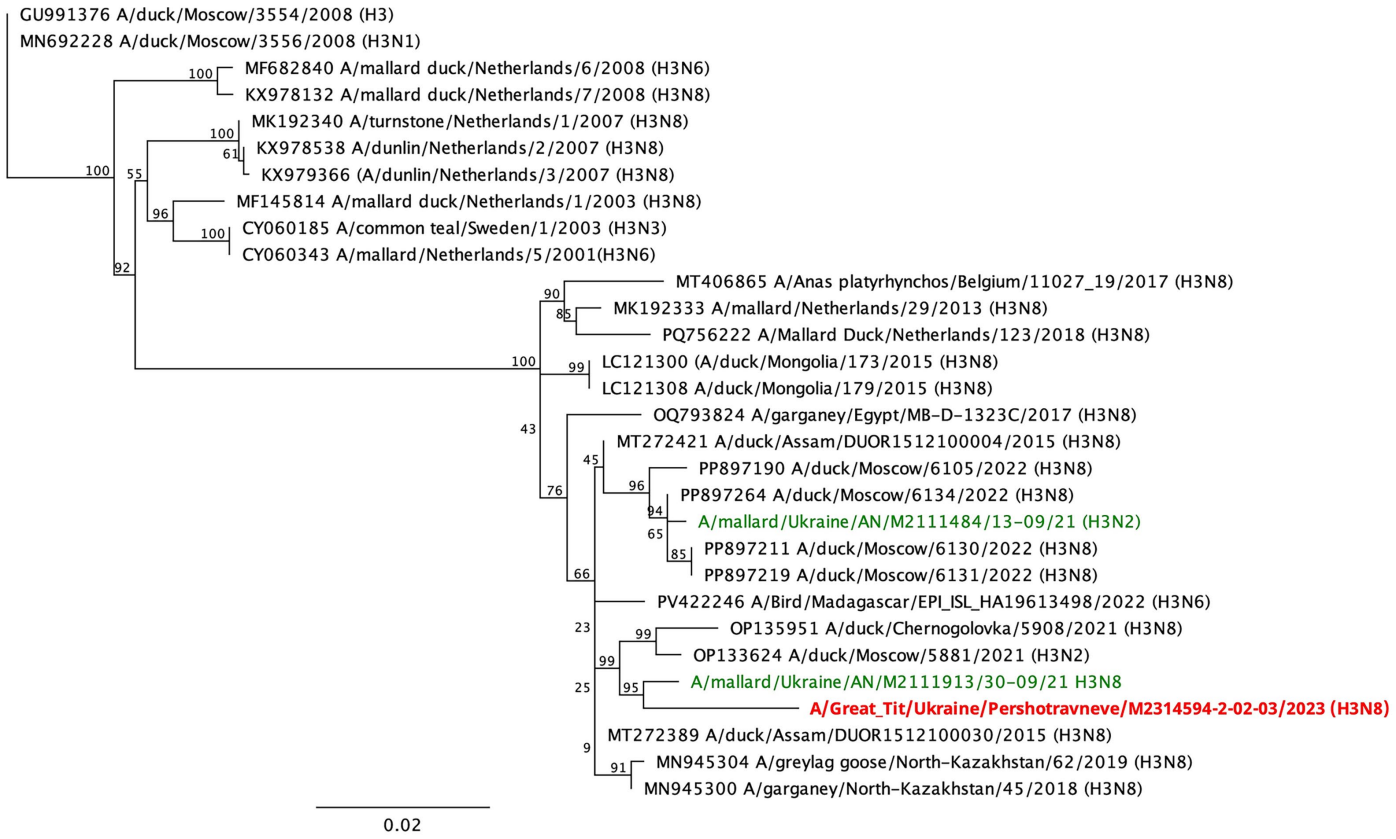
Phylogenetic tree constructed based on the results of sequencing the complete HA gene of LPAIV Н3N8 subtype detected in great tit in Ukraine (red color). Samples in green are other viruses the same subtype isolated from wild birds in Ukraine.

The influenza virus detected in Great Tit *Parus major* in Ukraine in 2023 was identified as a low pathogenic cleavage site (PEKQTR↓GLF) virus belonging to the Eurasian lineage of influenza A viruses. Phylogenetically, it clustered within a group of Eastern European H3N8 viruses (Figure 4). This cluster also includes another Ukrainian H3N8 virus previously isolated from wild waterfowl in the southern region of Ukraine in 2021, indicating a shared evolutionary background between viruses circulating in wild waterfowl and those detected in Passeriformes.

## 4 Discussion

The results of our 20-year-long serological, virological, and PCR surveillance of Passeriformes in Ukraine confirm a low-level circulation of influenza A virus among these birds. In total, 65 passerine species (of the 165 registered species in Ukraine), representing a variety of migratory statuses and ecological characteristics, were examined. The study encompassed diverse regions of Ukraine and included samples collected during different stages of the birds’ life cycle (migration, breeding, and wintering). Overall, only a small proportion of the tested birds showed the positive results for influenza A virus: 1.2% of blood serum samples, 8.9% of egg yolk samples, 0.15% from virus isolation, and 3.6% by PCR. Of the 20 families studied, only eight tested positive for influenza A virus: Corvidae (serologically), Emberizidae (serologically), Muscicapidae (serologically, virologically, PCR), Sylviidae (serologically, PCR), Paridae (serologically, yolks, PCR), Passeridae (serologically, yolks, PCR), Fringillidae (PCR), Panuridae (PCR). In our studies, conducted using different methods, we also obtained divergent results. Virological examinations showed a low positivity rate in Passeriformes (0.15%) compared to PCR results, where the proportion of positive samples reached 3.6%. In our view, there are several explanations for this. First, PCR is a more sensitive and specific method than virological techniques (Qian et al., 2025). In addition, virological studies detect only the circulation of viable influenza viruses capable of replication in laboratory biological systems. Similar discrepancies between the results of virological testing and PCR have also been reported by other researchers in wild birds (Qin et al., 2011; Kim et al., 2019). Given the fact that influenza viruses of subtypes H1, H3, and H7 are of epidemiological importance to human health, their monitoring is essential. Subtypes H1 and H3 cause seasonal influenza epidemics in humans; moreover, the first fatal human infection with an avian influenza H3N8 virus has recently been reported (Yang et al., 2022). Influenza viruses of the H7 subtype are also capable of causing human disease, ranging from conjunctivitis to severe pneumonia with fatal outcomes (Fouchier et al., 2004). Therefore, surveillance of these influenza virus subtypes, especially in new and atypical natural hosts, is of great importance. In our study, we did not identify any relationship between the H1, H3, and H7 influenza viruses circulating among Passeriformes in Ukraine and human influenza viruses of the same subtypes. Nevertheless, our findings highlight the need for continued research and more in-depth investigation of the genetic and antigenic characteristics of viruses circulating in atypical hosts.

Our findings are consistent with other studies indicating that certain Passeriformes families are more frequently associated with the potential transmission of influenza viruses (Williams et al., 2023). Williams R., summarizing data on multiple avian pathogens (including influenza), identified the following families as among the most frequently infected of them: Turdidae (which includes widespread and abundant species such as Common Blackbirds and European Robins), Estrildidae, Fringillidae (with many common species), Passeridae, Corvidae, Sturnidae, and Paridae, many of which are widely distributed and include synanthropic species (Callaghan et al., 2021). In the United States, during 2005–2008, a study of 22 passerine species (comprising over 4,000 samples) found 0.8% PCR-positive for AIVs (Fuller et al., 2010). Five species tested positive, with prevalence ranging from 9.0 to 50%, which is in line with our prevalence estimates for certain species. In China, a study conducted during 2005–2007 found that 2.3% of Passeriformes from the families Emberizidae, Motacillidae, Muscicapidae, Paridae, Passeridae, Sylviidae, Timaliidae, and Zosteropidae were AIV PCR-positive (Peterson et al., 2008). Among these, 4.8% samples from migratory species and 1.8% from non-migratory were positive. The difference in prevalence between open-habitat and forest birds was minimal: 2.9% vs. 2.4%, respectively. None of the tested samples were positive for the H5 subtype. In our study, seroprevalence was ranging from 1.96 to 12.5% per species However, according to some published sources, seroprevalence among synanthropic Passeriformes may reach 6–79% (Slusher et al., 2014; Hadipour et al., 2011). At the same time, other studies have reported no positive samples: for example, research conducted in Northern Europe between 1998 and 2006 involving over 4,500 Passeriformes from 16 families yielded no positive detections for influenza A virus (Munster et al., 2007).

Analyzing the results of our studies in Ukraine, we detected influenza virus antibodies in birds of the family Corvidae, which are widely distributed both globally and within Ukraine. These birds are generally omnivorous and are frequently observed near poultry and livestock farms (Winkler et al., 2020). Although previous studies (Shriner and Root, 2020) did not detect influenza antibodies in Corvidae, our findings revealed the presence of antibodies in a small number of individuals inhabiting areas in close proximity to poultry farms. At the same time, we did not detect the virus itself or any PCR-positive samples from Corvidae in our investigations. However, influenza virus infections (mainly HPAI H5) have been reported in Corvidae in India (Nagarajan et al., 2010), Bangladesh (Khan et al., 2014), and Germany (Globig et al., 2017). These findings support the certain involvement of Corvidae in the outbreaks of HPAI, although data on their role in the ecology of LPAI viruses remain limited (Ferrazzi et al., 2007).

In analyzing the obtained results, we will take a closer look at several species and families of Passeriformes and their ecological characteristics that may have epidemiological significance for the circulation of influenza A. We found that 1.42% of House Sparrows *Passer domesticus* were positive for AIV in PCR, and 10.0% had AIV antibodies in egg yolk samples. Positive individuals were detected in urban areas and near poultry farms. This species, along with the Eurasian Tree Sparrow *Passer montanus*, belongs to the Passeridae family and is a well-known synanthropic bird. Both species are commonly found in urbanized landscapes and frequently inhabit areas in close proximity to farms and human settlements. According to published data comprising 13 studies (Shriner and Root, 2020), the seroprevalence of these sparrows reaches 11.4%, while the PCR-based prevalence is significantly lower, at 0.6%. Additionally, a substantial number of experimental studies have demonstrated successful infections of sparrows with various subtypes of influenza viruses, including both low and highly pathogenic strains, confirming the ability of the virus to at least replicate in these birds (Shriner and Root, 2020). In this study, we detected influenza A virus in Passeriformes that are not typically considered synanthropic. Birds of the family Turdidae generally do not exhibit synanthropic behavior, yet they are commonly observed in urbanized landscapes and in proximity to poultry and livestock farms. In the United States, some Turdidae species (e.g., American Robins and Swainson’s Thrushes *Catharus ustulatus*) have been reported as seropositive for influenza A virus, with viral RNA also detected via PCR. (Shriner and Root, 2020) Furthermore, experimental infection studies have shown that members of this family are susceptible to HPAI viruses (Shriner and Root, 2020). In our studies conducted in Ukraine, we not only isolated two LPAI viruses (H7N1 and H1N1) from Fieldfare in 2021, but also detected PCR-positive samples from four additional Turdidae species: Common Blackbird, Collared Flycatcher, European Robin, and Song Thrush, confirming the presence of influenza viruses within this family. The family Fringillidae represents a large group of primarily non-synanthropic birds, though some individuals, such as the Eurasian Chaffinch, may display limited synanthropic behavior. There are relatively few reports of influenza detection in this group, some PCR-positive individuals have been reported in California (USA), whereas no positives were found in Germany (Shriner and Root, 2020). In contrast, in our research we identified three Fringillidae species as PCR-positive for influenza A virus, with prevalence rates ranging from 4.8 to 6.6%: European Chaffinch, European Goldfinch, and European Greenfinch.

In summary, while Passeriformes are not considered a new reservoir for influenza A viruses, they remain a poorly studied group, and current evidence suggests the need for further in-depth investigations. Our data clearly confirm the presence of influenza virus in wild passerine birds in Ukraine and underscore the importance of continuing and expanding surveillance to include non-waterfowl and non-aquatic ecological groups of wild birds. Moreover, our findings highlight the need to improve sampling methodologies, ensuring sufficient quantity and volume of materials (e.g., swabs, feces, blood samples) for testing, or to explore alternative sampling and diagnostic approaches, particularly for small-bodied bird species that are often excluded from monitoring due to sampling difficulties. In addition, the observed low seroprevalence in contrast to higher PCR detection rates indicates the necessity for further studies and/or improvements in serological diagnostic tools. While the detection of antibodies, viral isolates, and/or influenza viral RNA provides strong evidence for virus circulation, it does not yet elucidate the ecological dynamics of natural influenza virus circulation in Passeriformes. This gap in knowledge represents a promising direction for future research.

Study limitations. This article presents summarized results of study conducted using various methods over a 20-year period, which may impose certain limitations. First, the aim of the study was not to compare different methods, but rather to present the results obtained through these approaches. The choice of diagnostic methods was determined by the availability of resources, test-kits, and reagents at the time of researcher. Second, during field studies, only a small number of samples were obtained for certain species at some locations. In several cases these few samples tested positive (50–100% prevalence), making it impossible to perform robust statistical analyses on these data. Third, given that most of the birds are small-bodied species, the amount of biological material available for testing (feces/cloacal swabs) was limited. As a result, the quantity of viral RNA in PCR-positive samples was often low, which prevented performing full subtype identification by PCR and conducting whole-genome sequencing. At the same time, these limitations do not affect the overall results or the main conclusions of the study. Rather, they underscore the need for continued research on monitoring the natural circulation of influenza A viruses among atypical hosts, as well as for improving methods for detecting influenza antibodies and viral genetic material.

## Data Availability

The datasets presented in this study can be found in online repositories. The names of the repository/repositories and accession number(s) can be found at: https://www.ncbi.nlm.nih.gov/genbank/, PX091979; https://gisaid.org, EPI4242121.
