# Supplementary material for: Wild passerines as potential carriers and sources of avian influenza viruses in Ukraine
Source: Front Microbiol. 2026 Jan 20;16:1736454. doi: 10.3389/fmicb.2025.1736454 (PMC12864440; doi:10.3389/fmicb.2025.1736454)
Supplement: Supplementary file 1 [file Table_1.docx]

**Supplementary table**

Table S1. Biological material from wild birds (Passeriformes) collected in Ukraine in the period 2004−2025

| Bird species | Region | Location | Year | Samples, total | | | |
| --- | --- | --- | --- | --- | --- | --- | --- |
|  |  |  |  | Fecal/swab for virology (2010-2021) | Fecal/swab for PCR (2023-2025) | Serum  (2004-2025) | Eggs Yolk (2004-2025) |
| **Aegithalidae** | | | | | | | |
| Long-tailed Tit *Aegithalos caudatus* | Odesa Oblast | Ermakov | 2013 | 1 |  |  |  |
|  | Poltava Oblast | RLP Nyzhnovorsklianskyi | 2024 |  | 2 |  |  |
|  |  |  | 2025 |  | 3 |  |  |
|  | Lviv Oblast | NPP Yavorivskyi | 2024 |  | 11 |  |  |
| **Alaudidae** | | | | | | | |
| Calandra Lark *Melanocorypha calandra* | Kherson Oblast | Chongar | 2010 | 60 |  |  |  |
| Crested Lark *Galerida cristata* |  | Chaplynka | 2019 | 1 |  |  |  |
| **Bombycillidae** | | | | | | | |
| Waxwing *Bombycilla garrulus* | Kharkiv Oblast | Pershotravneve | 2023 |  | 10 |  |  |
| **Certhiidae** | | | | | | | |
| Treecreeper *Certhia familiaris* | Kyiv Oblast | Hlyboki Balyky | 2023 |  | 1 |  |  |
| **Corvidae** | | | | | | | |
| Rook *Corvus frugilegus* | Kharkiv region | Poultry farm №1 | 2004 |  |  | 1 |  |
|  | Odesa region | Odesa | 2005 |  |  |  | 8 |
|  |  |  | 2007 |  |  | 4 |  |
|  | Zaporizhzhia Oblast | Gyrsivka | 2011 | 7 |  |  |  |
|  |  | Dunaivka | 2011 | 16 |  |  |  |
|  | Odesa Oblast | Suhyi Lyman | 2012 | 165 |  |  |  |
|  |  | Prymorske | 2013 | 30 |  |  |  |
|  |  | Kilia | 2013 | 161 |  |  |  |
|  |  |  | 2019 | 4 |  |  |  |
|  | Kherson Oblast | Zaozerne | 2015 | 60 |  |  |  |
|  |  | Syvaske | 2019 | 1 |  |  |  |
|  |  | Vasylivka | 2019 | 2 |  |  |  |
|  |  | Chaplynka | 2019 | 5 |  |  |  |
|  |  | Askania-Nova | 2021 | 5 |  |  |  |
|  | Mykolaiv Oblast | Ochakiv | 2019 | 1 |  |  |  |
| Jackdaw *Coloeus monedula* | Kharkiv region | Poultry farm №2 | 2004 |  |  | 2 |  |
|  | Kherson Oblast | Chongar | 2011 | 15 |  |  |  |
|  |  | Zaozerne | 2015 | 10 |  |  |  |
|  |  | Askania-Nova | 2021 | 5 |  |  |  |
| Hooded Crow *Corvus cornix* | Zaporizhzhia region | Poultry farm №1 | 2004 |  |  | 4 |  |
|  | Kherson region | NNP «Dzharylhatskyi» | 2006 |  |  |  | 3 |
|  | Odesa Oblast | Sarata | 2019 | 1 |  |  |  |
|  |  | Reni | 2019 | 1 |  |  |  |
|  | Mykolaiv Oblast | Ochakiv | 2019 | 1 |  |  |  |
| Jay *Garrulus glandarius* | Poltava Oblast | Potoky | 2021 | 1 |  |  |  |
|  |  | RLP Nyzhnovorsklianskyi | 2023 |  | 1 | 1 |  |
|  |  |  | 2024 |  | 2 | 2 |  |
|  |  |  | 2025 |  | 4 | 2 |  |
|  | Kyiv Oblast | Hlyboki Balyky | 2023 |  | 2 | 2 |  |
|  | Kharkiv Oblast | Kharkiv (Hidropark) | 2006 |  |  |  | 1 |
|  |  | Pershotravneve | 2024 |  | 1 | 1 |  |
|  | Lviv Oblast | NPP Yavorivskyi | 2024 |  | 1 | - |  |
|  | Kirovograd Oblast | Ukrainka | 2025 |  |  |  | 1 |
| Magpie *Pica pica* | Kharkiv region | Kharkiv (Saltivka) | 2006 |  |  |  | 2 |
|  |  | Haidary | 2006 |  |  |  | 2 |
|  |  | Hineievka | 2006 |  |  |  | 5 |
|  |  | Vysokyi | 2006 |  |  |  | 1 |
|  | Kherson region | NNP «Dzharylhatskyi» | 2006 |  |  |  | 3 |
|  | AR Crimea | Ermakove | 2011 | 35 |  |  |  |
|  | Odesa Oblast | Ermakiv | 2013 | 2 |  |  |  |
|  | Mykolaiv Oblast | Ochakiv | 2019 | 1 |  |  |  |
| **Emberizidae** | | | | | | | |
| Reed Bunting *Emberiza schoeniclus* | Kherson Oblast | Chongar | 2010 |  | 5 |  |  |
|  | AR Crimea | Tup-Tarchan | 2011 |  | 15 |  |  |
|  | Zaporizhzhia Oblast | Utluk | 2011 |  | 1 |  |  |
|  | Odesa Oblast | Ermakiv | 2013 |  | 1 |  |  |
|  |  | NPP Tuzlivski Lymany | 2024 |  |  | 10 |  |
| Yellowhammer *Emberiza citrinella* | Poltava Oblast | RLP Nyzhnovorsklianskyi | 2023 |  | 3 | 1 |  |
|  |  |  | 2024 |  | 6 | 3 |  |
|  |  |  | 2025 |  | 2 | 2 |  |
|  | Kirovograd Oblast | Ukrainka | 2025 |  |  |  | 1 |
| Corn Bunting *Emberiza calandra* | Odesa Oblast | Prymorske | 2013 | 4 |  |  |  |
| **Fringillidae** | | | | | | | |
| Chaffinch *Fringilla coelebs* | Kharkiv Oblast | Hineievka | 2006 |  |  |  | 3 |
|  |  | Gaidary | 2019 | 18 |  |  |  |
|  |  |  | 2023 |  | 22 | 3 |  |
|  |  | Pershotravneve | 2024 |  | 5 | 6 |  |
|  |  |  | 2025 |  |  | 3 |  |
|  | Khmelnytska Oblast | Maliivtsi | 2023 |  | 5 |  | 1 |
|  | Poltava Oblast | RLP Nyzhnovorsklianskyi | 2024 |  | 17 | 6 |  |
|  |  |  | 2025 |  | 8 | 2 |  |
|  | Kherson Oblast | Chongar | 2010 | 1 |  |  |  |
|  |  |  | 2011 | 1 |  |  |  |
| Goldfinch *Carduelis carduelis* | Odesa Oblast | Ermakiv | 2013 | 23 |  |  |  |
|  | Kharkiv Oblast | Gaidary | 2023 |  | 6 | 1 |  |
|  | Poltava Oblast | RLP Nyzhnovorsklianskyi | 2023 |  | 6 | 6 |  |
|  |  |  | 2024 |  | 2 |  |  |
|  |  |  | 2025 |  | 1 |  |  |
| Greenfinch *Chloris chloris* | Kharkiv Oblast | Gaidary | 2006 |  |  |  | 1 |
|  |  |  | 2023 |  | 8 | 1 |  |
|  | Khmelnytska Oblast | Maliivtsi | 2023 |  | 2 |  |  |
|  | Poltava Oblast | RLP Nyzhnovorsklianskyi | 2023 |  | 43 | 33 | 3 |
|  |  |  | 2024 |  | 21 | 9 |  |
|  |  |  | 2025 |  | 30 | 18 |  |
|  | Sumy region | NNP «Hetmanskyi» | 2006 |  |  |  | 1 |
| Hawfinch *Coccothraustes coccothraustes* | Kharkiv Oblast | Gaidary | 2006 |  |  |  | 3 |
|  |  |  | 2023 |  | 11 | 8 |  |
|  |  | Pershotravneve | 2024 |  | 1 | 1 |  |
|  |  |  | 2025 |  |  | 1 |  |
|  | Khmelnytska Oblast | Maliivtsi | 2023 |  | 2 |  |  |
|  | Poltava Oblast | RLP Nyzhnovorsklianskyi | 2023 |  | 3 | 3 |  |
|  |  |  | 2024 |  | 3 | 5 |  |
|  |  |  | 2025 |  | 7 | 5 |  |
|  | Kirovohradska Oblast | Ukrainka | 2025 |  | 1 |  |  |
| **Hirundinidae** | | | | | | | |
| House Martin *Delichon urbica* | Poltava Oblast | RLP Nyzhnovorsklianskyi | 2023 |  | 15 | 7 |  |
| Swallow *Hirundo rustica* | Kharkiv region | Poultry farm №1 | 2004 |  |  | 1 |  |
|  |  |  | 2004 |  |  |  | 6 |
|  |  | Poultry farm №3 | 2004 |  |  |  | 10 |
|  | Zaporizhzhia Oblast | Davydivka | 2007 |  |  | 6 |  |
|  |  |  | 2019 | 2 |  |  |  |
|  |  | Utluk | 2011 | 8 |  |  |  |
|  | Poltava Oblast | RLP Nyzhnovorsklianskyi | 2023 |  | 18 | 12 |  |
|  | Odesa Oblast | Ermakiv | 2013 | 2 |  |  |  |
|  | Kherson Oblast | Kairka | 2019 | 1 |  |  |  |
| Sand Martin *Riparia riparia* | Zaporizhzhia Oblast | Davydivka | 2007 |  |  | 2 |  |
|  |  | Utluk | 2011 | 3 |  |  |  |
|  |  | Ochrymivka | 2015 | 10 |  |  |  |
|  |  |  | 2018 | 124 |  |  |  |
|  |  |  | 2019 | 117 |  |  |  |
| **Laniidae** | | | | | | | |
| Lesser Grey Shrike *Lanius minor* | Poltava Oblast | Luchky, RLP «Nyzhnovorsklianskyi» | 2024 |  | 1 |  |  |
| Red-backed Shrike *Lanius collurio* | Kharkiv region | Haidary | 2006 |  |  |  | 3 |
|  |  | Zanky | 2006 |  |  |  | 1 |
|  |  | Hineievka | 2006 |  |  |  | 1 |
|  | Poltava Oblast | Luchky, RLP «Nyzhnovorsklianskyi» | 2023 |  | 9 | 5 |  |
| **Motacillidae** | | | | | | | |
| Pied Wagtail *Motacilla alba* | Zaporizhzhia Oblast | Utluk | 2011 | 1 |  |  |  |
|  | Odesa Oblast | Ermakiv | 2013 | 9 |  |  |  |
|  | Poltava Oblast | RLP Nyzhnovorsklianskyi | 2023 |  | 1 | 1 |  |
|  |  |  | 2024 |  | 1 | 1 |  |
|  |  |  | 2025 |  | 1 |  |  |
| Tree Pipit *Anthus trivialis* | Kirovohradska Oblast | Ukrainka | 2025 |  | 1 |  |  |
| Yellow Wagtail *Motacilla flava* | Zaporizhzhia Oblast | Utluk | 2011 | 1 |  |  |  |
|  | Odesa Oblast | Trapivka | 2024 |  | 1 |  |  |
| **Muscicapidae** | | | | | | | |
| Black Redstart *Phoenicurus ochruros* | Kharkiv Oblast | Gaidary | 2006 |  |  |  | 1 |
|  |  |  | 2023 |  | 1 |  |  |
|  | Lviv Oblast | NPP Yavorivskyi | 2024 |  | 1 |  |  |
| Blackbird *Turdus merula* | Kharkiv Oblast | Gaidary | 2006 |  |  |  | 4 |
|  |  |  | 2023 |  | 11 | 7 | 1 |
|  |  | Hineievka | 2006 |  |  |  | 2 |
|  |  | Pershotravneve | 2024 |  | 9 | 9 |  |
|  |  |  | 2025 |  |  | 1 |  |
|  | Khmelnytska Oblast | Maliivtsi | 2023 |  | 6 | 1 | 1 |
|  |  | NPP «Podilski  Tovtry» | 2024 |  | 1 | 1 |  |
|  | Poltava Oblast | RLP Nyzhnovorsklianskyi | 2023 |  | 11 | 10 |  |
|  |  |  | 2024 |  | 12 | 14 |  |
|  |  |  | 2025 |  | 10 | 6 |  |
|  | Kyiv Oblast | Hlyboki Balyky | 2023 |  | 2 | 2 |  |
|  | Kirovohradska Oblast | Ukrainka | 2025 |  | 3 |  | 1 |
| Bluethroat *Luscinia svecica* | Kharkiv region | Gaidary | 2007 |  |  |  | 1 |
|  | Poltava Oblast | RLP Nyzhnovorsklianskyi | 2023 |  | 1 |  |  |
| Collared Flycatcher *Ficedula albicollis* | Sumy region | Vakalivshchyna, | 2004 |  |  |  | 3 |
|  |  | NNP «Hetmanskyi» | 2006 |  |  |  | 9 |
|  | Khmelnytska Oblast | Maliivtsi | 2023 |  | 4 |  |  |
|  | Odesa Oblast | Lyman | 2024 |  | 5 | 2 |  |
|  |  | Trapivka-2 | 2024 |  | 2 | 2 |  |
|  | Kharkiv region | Haidary | 2006 |  |  |  | 11 |
|  |  | RLP «Feldman ecopark» | 2006 |  |  |  | 9 |
|  |  |  | 2021 | 4 |  |  |  |
|  |  | NNP «Homilshanski lisy» | 2006 |  |  |  | 6 |
|  |  | Hineievka | 2006 |  |  |  | 2 |
| Pied Flycatcher *Ficedula hypoleuca* |  | Haidary | 2007 |  |  |  | 4 |
|  |  | NNP «Homilshanski lisy» | 2007 |  |  |  | 4 |
|  | Poltava Oblast | RLP Nyzhnovorsklianskyi | 2024 |  | 1 |  |  |
|  |  |  | 2025 |  | 1 |  |  |
|  | Kirovohradska Oblast | Ukrainka | 2025 |  | 1 |  |  |
| Robin *Erithacus rubecula* | Kharkiv Oblast | Gaidary | 2006 |  |  |  | 1 |
|  |  |  | 2023 |  | 10 | 1 |  |
|  |  | Vasyschevo | 2020 | 17 |  |  |  |
|  |  | Pershotravneve | 2024 |  | 4 | 1 |  |
|  | Poltava Oblast | Potoky | 2021 | 1 |  |  |  |
|  |  | RLP Nyzhnovorsklianskyi | 2023 |  | 1 |  |  |
|  |  |  | 2024 |  | 24 | 10 |  |
|  |  |  | 2025 |  | 17 |  |  |
|  | Kyiv Oblast | Hlyboki Balyky | 2023 |  | 9 |  |  |
|  | Khmelnytska Oblast | NPP «Podilski  Tovtry» | 2024 |  | 6 |  |  |
|  | Lviv Oblast | Yavorivskyi NPP | 2024 |  | 3 |  |  |
|  | Sumy region | NNP «Hetmanskyi» | 2006 |  |  |  | 9 |
| Song Thrush *Turdus philomelos* | Kharkiv Oblast | Gaidary | 2006 |  |  |  | 11 |
|  |  |  | 2019 | 1 |  |  |  |
|  |  |  | 2023 |  | 17 | 7 |  |
|  |  | Vasyschevo | 2020 | 1 |  |  |  |
|  |  | Kharkiv (Hydropark) | 2006 |  |  |  | 1 |
|  |  | NNP «Homilshanski lisy» | 2006 |  |  |  | 4 |
|  |  |  | 2007 |  |  |  | 1 |
|  |  | Pershotravneve | 2024 |  | 7 | 5 |  |
|  |  |  | 2025 |  |  | 1 |  |
|  | Poltava Oblast | Potoky | 2021 | 5 |  |  |  |
|  |  | RLP Nyzhnovorsklianskyi | 2023 |  | 13 | 10 | 1 |
|  |  |  | 2024 |  | 21 | 21 |  |
|  |  |  | 2025 |  | 7 | 3 |  |
|  | Khmelnytska Oblast | Maliivtsi | 2023 |  |  |  | 1 |
|  |  | NPP «Podilski Tovtry» | 2024 |  | 1 | 1 |  |
|  | Odesa Oblast | NPP Tuzlivski Lymany | 2024 |  |  | 1 |  |
|  | Sumy region | NNP «Hetmanskyi» | 2006 |  |  |  | 2 |
|  |  | Vakalivshchyna | 2006 |  |  |  | 1 |
| Spotted Flycatcher *Muscicapa striata* | Kharkiv Oblast | Gaidary | 2019 | 1 |  |  |  |
|  | Poltava Oblast | RLP Nyzhnovorsklianskyi | 2023 |  | 3 | 2 |  |
|  |  |  | 2024 |  | 1 | 1 |  |
| Stonechat *Saxicola torquata* | Poltava Oblast | RLP Nyzhnovorsklianskyi | 2024 |  | 1 | 2 |  |
| Thrush Nightingale *Luscinia luscinia* | Poltava Oblast | RLP Nyzhnovorsklianskyi | 2023 |  | 2 | 2 |  |
|  |  |  | 2024 |  | 1 |  |  |
|  | Odesa Oblast | Liman | 2024 |  | 1 |  |  |
|  |  | Trapivka-2 | 2024 |  | 1 | 1 |  |
| Whinchat *Saxicola rubetra* | Poltava Oblast | RLP Nyzhnovorsklianskyi | 2023 |  | 1 |  |  |
| Fieldfare *Turdus pilaris* | Kharkiv region | Kharkiv (Hydropark) | 2006 |  |  |  | 6 |
|  |  | Hineievka | 2006 |  |  |  | 3 |
|  | Zaporizhzhia Oblast | Bogatyr | 2021 | 18 |  |  |  |
| **Oriolidae** | | | | | | | |
| Golden Oriole *Oriolus oriolus* | Poltava Oblast | RLP Nyzhnovorsklianskyi | 2023 |  | 4 | 4 |  |
| **Panuridae** | | | | | | | |
| Bearded Tit *Panurus biarmicus* | Zaporizhzhia Oblast | Utluk | 2011 | 8 |  |  |  |
|  | Odesa Oblast | NPP Tuzlivski Lymany | 2024 |  | 2 | 2 |  |
| **Paridae** | | | | | | | |
| Blue Tit *Parus caeruleus* | Kyiv Oblast | Hlyboki Balyky | 2023 |  | 5 |  |  |
|  | Kharkiv Oblast | RLP «Feldman ecopark» | 2006 |  |  |  | 2 |
|  |  | Gaidary | 2019 | 1 |  |  |  |
|  |  |  | 2023 |  | 3 |  |  |
|  |  | Vasyschevo | 2020 | 3 |  |  |  |
|  |  | Pershotravneve | 2023 |  | 5 |  |  |
|  |  |  | 2024 |  | 2 |  |  |
|  | Poltava Oblast | RLP Nyzhnovorsklianskyi | 2024 |  | 3 |  |  |
|  |  |  | 2025 |  | 4 | 1 |  |
|  | Khmelnytska Oblast | NPP «Podilski  Tovtry» | 2024 |  | 2 |  |  |
| Great Tit *Parus major* | Kharkiv Oblast | RLP «Feldman ecopark» | 2006 |  |  |  | 1 |
|  |  | NNP «Homilshanski lisy» | 2006 |  |  |  | 14 |
|  |  | Dergachi | 2022 |  | 2 |  |  |
|  |  | Manchenki | 2022 |  | 55 |  |  |
|  |  | Pershotravneve | 2023 |  | 22 | 11 |  |
|  |  |  | 2024 |  | 14 | 15 |  |
|  |  |  | 2025 |  |  | 8 |  |
|  |  | Gaidary | 2019 | 2 |  |  |  |
|  |  |  | 2023 |  | 29 |  |  |
|  |  | Vasyschevo | 2020 | 1 |  |  |  |
|  | Poltava Oblast | RLP Nyzhnovorsklianskyi | 2023 |  | 13 | 7 |  |
|  |  |  | 2024 |  | 16 | 1 |  |
|  |  |  | 2025 |  | 23 |  |  |
|  |  | Horishni Plavni | 2023 |  |  |  | 1 |
|  | Kyiv Oblast | Hlyboki Balyky | 2023 |  | 15 | 8 |  |
|  | Lviv Oblast | NPP Yavorivskyi | 2024 |  | 17 |  |  |
|  | Odesa Oblast | Liman | 2024 |  | 2 | 1 |  |
|  |  | Trapivka | 2024 |  | 1 | 1 |  |
|  |  | Trapivka-2 | 2024 |  | 5 | 2 |  |
|  | Khmelnytska Oblast | NPP «Podilski  Tovtry» | 2024 |  | 2 |  |  |
|  | Kirovohradska Oblast | Ukrainka | 2025 |  | 1 |  |  |
|  | Sumy region | NNP «Hetmanskyi» | 2006 |  |  |  | 7 |
|  |  | Vakalivshchyna | 2006 |  |  |  | 2 |
| Marsh Tit *Parus palustris* | Kharkiv Oblast | NNP «Homilshanski lisy» | 2006 |  |  |  | 3 |
|  |  | Gaidary | 2019 | 3 |  |  |  |
|  |  |  | 2023 |  | 1 |  |  |
|  |  | Pershotravneve | 2024 |  | 3 |  |  |
|  |  | Vasyschevo | 2020 | 1 |  |  |  |
|  | Kyiv Oblast | Hlyboki Balyky | 2023 |  | 3 |  |  |
|  | Khmelnytska Oblast | NPP «Podilski  Tovtry» | 2024 |  | 3 |  |  |
|  | Lviv Oblast | NPP Yavorivskyi | 2024 |  | 1 |  |  |
| **Passeridae** | | | | | | | |
| House Sparrow *Passer domesticus* | Kharkiv Oblast | Poultry farm №1 | 2004 |  |  | 2 | 40 |
|  |  | Poultry farm №2 | 2004 |  |  | 12 |  |
|  |  | Poultry farm №3 | 2004 |  |  |  | 29 |
|  |  | Poultry farm №4 | 2004 |  |  | 10 |  |
|  |  | Dergachi | 2022 |  | 33 |  |  |
|  |  | Manchenki | 2022 |  | 29 |  |  |
|  |  | Pershotravneve | 2023 |  | 8 |  |  |
|  |  |  | 2024 |  |  | 1 |  |
|  |  |  | 2025 |  |  | 1 |  |
|  | Odesa Oblast | Prymorske | 2012 | 52 |  |  |  |
|  | Mykolaiv Oblast | Ochakiv | 2019 | 1 |  |  |  |
| Tree Sparrow *Passer montanus* | Poltava Oblast | RLP Nyzhnovorsklianskyi | 2023 |  | 29 | 14 |  |
|  |  |  | 2024 |  | 12 | 2 |  |
|  |  |  | 2025 |  | 24 | 7 |  |
|  | Odesa Oblast | Liman | 2024 |  | 1 | 1 |  |
|  |  | Trapivka-2 | 2024 |  | 3 | 5 |  |
|  | Kherson Oblast | Novyi Trud | 2012 | 5 |  |  |  |
|  | Kharkiv Oblast | Gaidary | 2019 | 1 |  |  |  |
| Spanish Sparrow *Passer hispaniolensis* | Odesa Oblast | Trapivka | 2024 |  |  | 3 |  |
|  |  | Trapivka-2 | 2024 |  |  | 3 |  |
| **Regulidae** | | | | | | | |
| Goldcrest *Regulus regulus* | Lviv Oblast | Yavorivskyi NPP | 2024 |  | 12 |  |  |
| **Sittidae** | | | | | | | |
| Nuthatch *Sitta europaea* | Khmelnytska Oblast | Maliivtsi | 2023 |  | 1 |  |  |
|  | Kyiv Oblast | Hlyboki Balyky | 2023 |  | 1 |  |  |
|  | Lviv Oblast | NPP Yavorivskyi | 2024 |  | 2 |  |  |
|  | Kharkiv Oblast | Pershotravneve | 2025 |  |  | 1 |  |
| **Sturnidae** | | | | | | | |
| Starling *Sturnus vulgaris* | AR Crimea | Medvedivla | 2010 | 35 |  |  |  |
|  |  | Istochne | 2012 | 91 |  |  |  |
|  | Zaporizhzhia Region, | Davydivka | 2007 |  |  | 1 |  |
|  |  | Utluk | 2011 | 2 |  |  |  |
|  |  | Gyrsivka | 2015 | 25 |  |  |  |
|  | Kharkiv Oblast | Dergachi | 2022 |  | 47 |  |  |
|  | Poltava Oblast | RLP Nyzhnovorsklianskyi | 2023 |  | 1 | 1 |  |
|  | Odesa Oblast | NPP Tuzlivski Lymany | 2024 |  |  | 2 |  |
|  |  | Tarutynskyy step | 2024 |  |  | 1 |  |
|  |  | Reni | 2019 | 6 |  |  |  |
|  | Kherson Oblast | Chongar | 2016 | 25 |  |  |  |
|  | Mykolaiv Oblast | Ochakiv | 2019 | 3 |  |  |  |
| Sylviidae | | | | | | | |
| Barred Warbler *Sylvia nisoria* | Poltava Oblast | RLP Nyzhnovorsklianskyi | 2023 |  | 4 | 2 |  |
| Blackcap *Sylvia atricapilla* | Sumy region | Vakalivshchyna | 2006 |  |  |  | 2 |
|  | Khmelnytska Oblast | Maliivtsi | 2023 |  | 2 |  |  |
|  |  | NPP «Podilski  Tovtry» | 2024 |  | 4 | 1 |  |
|  | Poltava Oblast | RLP Nyzhnovorsklianskyi | 2023 |  | 6 |  |  |
|  |  |  | 2024 |  | 4 | 2 |  |
|  |  |  | 2025 |  | 8 |  |  |
|  | Odesa Oblast | Trapivka | 2024 |  |  | 1 |  |
|  |  | Lyman | 2024 |  | 1 | 1 |  |
|  |  | Trapivka-2 | 2024 |  | 3 | 3 |  |
|  | Kirovohradska Oblast | Ukrainka | 2025 |  | 2 |  | 1 |
|  | Kharkiv Oblast | Gaidary | 2019 | 4 |  |  |  |
|  |  | Kharkiv (Feldman Ecopark) | 2021 | 1 |  |  |  |
| Chiffchaff *Phylloscopus collybita* | Poltava Oblast | RLP Nyzhnovorsklianskyi | 2023 |  | 2 |  |  |
|  |  |  | 2024 |  | 4 |  |  |
|  |  |  | 2025 |  | 10 |  |  |
|  | Kharkiv Oblast | Gaidary | 2019 | 1 |  |  |  |
|  |  | Pershotravneve | 2024 |  | 1 |  |  |
|  | Odesa Oblast | NPP Tuzlivski Lymany | 2024 |  | 1 | 1 |  |
|  | Kirovohradska Oblast | Ukrainka | 2025 |  | 1 |  |  |
| Great Reed Warbler *Acrocephalus arundinaceus* | Zaporizhzhia Region | Davydivka | 2007 |  |  | 5 |  |
|  |  | Utluk | 2011 | 11 |  |  |  |
|  | Poltava Oblast | RLP Nyzhnovorsklianskyi | 2023 |  | 4 | 4 |  |
|  | Odesa Oblast | Liman | 2024 |  | 2 | 3 |  |
|  |  | Trapivka | 2024 |  | 1 | 1 |  |
|  |  | Trapivka-2 | 2024 |  | 1 | 1 |  |
| Icterine Warbler *Hippolais icterina* | Odesa Oblast | Trapivka-2 | 2024 |  | 1 |  |  |
|  | Zaporizhzhia Oblast | Utluk | 2011 | 1 |  |  |  |
| Lesser Whitethroat *Sylvia curruca* | Odesa Oblast | Ermakiv | 2013 | 3 |  |  |  |
|  |  | Liman | 2024 |  | 9 |  |  |
|  |  | Trapivka | 2024 |  | 11 |  |  |
|  |  | Trapivka-2 | 2024 |  | 4 |  |  |
|  | Poltava Oblast | RLP Nyzhnovorsklianskyi | 2025 |  | 1 |  |  |
| Marsh Warbler  *Acrocephalus palustris* | Odesa Oblast | Lyman | 2024 |  | 1 | 1 |  |
| Paddyfield Warbler *Acrocephalus agricola* | Odesa Oblast | Lyman | 2024 |  | 6 | 2 |  |
| Reed Warbler *Acrocephalus scirpaceus* | Zaporizhzhia Oblast | Utluk | 2011 | 4 |  |  |  |
|  | Odesa Oblast | Liman | 2024 |  | 16 | 2 |  |
|  |  | Trapivka | 2024 |  | 2 | 1 |  |
|  |  | Trapivka-2 | 2024 |  | 1 |  |  |
| River Warbler *Locustella fluviatilis* | Odesa Oblast | NPP Tuzlivski Lymany | 2024 |  |  | 1 |  |
| Savi`s Warbler *Locustella luscinioides* | Zaporizhzhia Oblast | Utluk | 2011 | 1 |  |  |  |
|  | Odesa Oblast | Liman | 2024 |  | 1 |  |  |
| Sedge Warbler *Acrocephalus schoenobaenus* | Zaporizhzhia Oblast | Utluk | 2011 | 1 |  |  |  |
|  | Odesa Oblast | NPP Tuzlivski Lymany | 2024 |  | 2 | 1 |  |
|  |  | Lyman | 2024 |  |  | 1 |  |
| Whitethroat *Sylvia communis* | Poltava Oblast | RLP Nyzhnovorsklianskyi | 2023 |  | 9 | 2 |  |
|  | Odesa Oblast | Trapivka | 2024 |  | 3 | 3 |  |
|  |  | Lyman | 2024 |  |  | 2 |  |
|  |  | Trapivka-2 | 2024 |  |  | 1 |  |
| Willow Warbler *Phylloscopus trochilus* | Odesa Oblast | Liman | 2024 |  | 4 |  |  |
|  |  | Trapivka | 2024 |  | 3 |  |  |
|  |  | Trapivka-2 | 2024 |  | 1 |  |  |
| Olivaceous Warbler *Hippolais pallida* | Zaporizhzhia Oblast | Utluk | 2011 | 1 |  |  |  |
| Wood Warbler *Phylloscopus sibilatrix* | Kharkiv Oblast | Gaidary | 2019 | 1 |  |  |  |
| Troglodytidae | | | | | | | |
| Wren *Troglodytes troglodytes* | Poltava Oblast | RLP Nyzhnovorsklianskyi | 2024 |  | 1 |  |  |
|  | Lviv Oblast | Yavorivskyi NPP | 2024 |  | 2 |  |  |
