# Supplementary material for: Wild passerines as potential carriers and sources of avian influenza viruses in Ukraine
Source: Front Microbiol. 2026 Jan 20;16:1736454. doi: 10.3389/fmicb.2025.1736454 (PMC12864440; doi:10.3389/fmicb.2025.1736454)
Supplement: Supplementary file 2 [file Table_2.docx]

**Supplementary table**

Table S2. Results of seroprevalence to AIV in Passerines (in serum and eggs yolk) in Ukraine in 2004-2025 (full data including positive and negative results).

| Bird species | Region | Location | Year | Samples, total | Test type | Result | | Seroprev, % |
| --- | --- | --- | --- | --- | --- | --- | --- | --- |
|  |  |  |  |  |  | Pos | Neg |  |
| **Serum** | | | | | | | | |
| **Corvidae** | | | | | | | | |
| Rook *Corvus frugilegus* | Kharkiv region | Poultry farm №1, | 2004 | 1 | Н1-Н14 | 0 | 1 | - |
|  | Odesa region | Odesa | 2007 | 4 | Н1-Н14 | 0 | 4 | - |
| Jackdaw *Coloeus monedula* | Kharkiv region | Poultry farm №2, | 2004 | 2 | Н1-Н14 | 0 | 2 | - |
| Hooded Crow *Corvus cornix* | Zaporizhzhia region | Poultry farm №1 | 2004 | 4 | Н1-Н14 | 1 | 3 | 25.0 |
| Jay *Garrulus glandarius* | Poltava Oblast | RLP Nizhnevorsklyanskiy | 2023 | 1 | ELISA | 0 | 1 | 0 |
|  |  |  | 2024 | 2 | ELISA | 0 | 2 | 0 |
|  |  |  | 2025 | 2 | ELISA | 1 | 1 | 50.0 |
|  | Kyiv Oblast | Balyko-Schynsenka | 2023 | 2 | ELISA | 0 | 2 | 0 |
|  | Kharkiv Oblast | Pershotravneve | 2024 | 1 | ELISA | 0 | 1 | 0 |
| **Emberizidae** | | | | | | | | |
| Reed Bunting *Emberiza schoeniclus* | Odesa Oblast | NPP Tuzlivski Lymany | 2024 | 10 | ELISA | 1 | 9 | 10.0 |
| Yellowhammer *Emberiza citrinella* | Poltava Oblast | RLP Nizhnevorsklyanskiy | 2023 | 1 | ELISA | 0 | 1 | 0 |
|  |  |  | 2024 | 3 | ELISA | 0 | 3 | 0 |
|  |  |  | 2025 | 2 | ELISA | 0 | 2 | 0 |
| **Fringillidae** | | | | | | | | |
| Chaffinch *Fringilla coelebs* | Kharkiv Oblast | Gaidary | 2023 | 3 | ELISA | 0 | 3 | 0 |
|  | Kharkiv Oblast | Pershotravneve | 2024 | 6 | ELISA | 0 | 6 | 0 |
|  |  |  | 2025 | 3 | ELISA | 0 | 3 | 0 |
|  | Poltava Oblast | RLP Nizhnevorsklyanskiy | 2024 | 6 | ELISA | 0 | 6 | 0 |
|  |  |  | 2025 | 2 | ELISA | 0 | 2 | 0 |
| Goldfinch *Carduelis carduelis* | Kharkiv Oblast | Gaidary | 2023 | 1 | ELISA | 0 | 1 | 0 |
|  | Poltava Oblast | RLP Nizhnevorsklyanskiy | 2023 | 6 | ELISA | 0 | 6 | 0 |
| Greenfinch *Chloris chloris* | Kharkiv Oblast | Gaidary | 2023 | 1 | ELISA | 0 | 1 | 0 |
|  | Poltava Oblast | RLP Nizhnevorsklyanskiy | 2023 | 33 | ELISA | 0 | 33 | 0 |
|  |  |  | 2024 | 9 | ELISA | 0 | 9 | 0 |
|  |  |  | 2025 | 18 | ELISA | 0 | 18 | 0 |
| Hawfinch *Coccothraustes coccothraustes* | Kharkiv Oblast | Gaidary | 2023 | 8 | ELISA | 0 | 8 | 0 |
|  | Kharkiv Oblast | Pershotravneve | 2024 | 1 | ELISA | 0 | 1 | 0 |
|  |  |  | 2025 | 1 | ELISA | 0 | 1 | 0 |
|  | Poltava Oblast | RLP Nizhnevorsklyanskiy | 2023 | 3 | ELISA | 0 | 3 | 0 |
|  |  |  | 2024 | 5 | ELISA | 0 | 5 | 0 |
|  |  |  | 2025 | 5 | ELISA | 0 | 5 | 0 |
| **Hirundinidae** | | | | | | | | |
| Swallow *Hirundo rustica* | Kharkiv region | Poultry farm №1, | 2004 | 1 | Н1-Н14 | 0 | 1 | - |
|  | Zaporizhzhia Region | Davydivka | 2007 | 6 | Н1-Н14 | 0 | 6 | - |
|  | Poltava Oblast | RLP Nizhnevorsklyanskiy | 2023 | 12 | ELISA | 0 | 12 | 0 |
| Sand Martin *Riparia riparia* | Zaporizhzhia Region, | Davydivka | 2007 | 2 | Н1-Н14 | 0 | 2 | - |
| House Martin *Delichon urbica* | Poltava Oblast | RLP Nizhnevorsklyanskiy | 2023 | 7 | ELISA | 0 | 7 |  |
| **Laniidae** | | | | | | | | |
| Red-backed Shrike *Lanius collurio* | Poltava Oblast | RLP Nizhnevorsklyanskiy | 2023 | 5 | ELISA | 0 | 5 | 0 |
| **Motacillidae** | | | | | | | | |
| Pied Wagtail *Motacilla alba* | Poltava Oblast | RLP Nizhnevorsklyanskiy | 2023 | 1 | ELISA | 0 | 1 | 0 |
| Yellow Wagtail *Motacilla flava* | Odesa Oblast | Trapivka | 2024 | 1 | ELISA | 0 | 1 | 0 |
| **Muscicapidae** | | | | | | | | |
| Blackbird *Turdus merula* | Kharkiv Oblast | Gaidary | 2023 | 7 | ELISA | 0 | 7 | 0 |
|  |  | Pershotravneve | 2024 | 9 | ELISA | 0 | 9 | 0 |
|  |  |  | 2025 | 1 | ELISA | 0 | 1 | 0 |
|  | Khmelnytska Oblast | Maliivtsi | 2023 | 1 | ELISA | 0 | 1 | 0 |
|  |  | NNP «Podilski Tovtry» | 2024 | 1 | ELISA | 0 | 1 | 0 |
|  | Poltava Oblast | RLP Nizhnevorsklyanskiy | 2023 | 10 | ELISA | 1 | 9 | 10.0 |
|  |  |  | 2024 | 14 | ELISA | 0 | 14 | 0 |
|  |  |  | 2025 | 6 | ELISA | 0 | 6 | 0 |
|  | Kyiv Oblast | Balyko-Shchuchinka | 2023 | 2 | ELISA | 0 | 2 | 0 |
| Collared Flycatcher *Ficedula albicollis* | Odesa Oblast | Lyman | 2024 | 2 | ELISA | 0 | 2 | 0 |
|  |  | Trapivka-2 | 2024 | 2 | ELISA | 0 | 2 | 0 |
| Robin *Erithacus rubecula* | Kharkiv Oblast | Gaidary | 2023 | 1 | ELISA | 0 | 1 | 0 |
|  |  | Pershotravneve | 2024 | 1 | ELISA | 0 | 1 | 0 |
|  | Poltava Oblast | RLP Nizhnevorsklyanskiy | 2024 | 10 | ELISA | 0 | 10 | 0 |
| Song Thrush *Turdus philomelos* | Kharkiv Oblast | Gaidary | 2023 | 7 | ELISA | 0 | 7 | 0 |
|  |  | Pershotravneve | 2024 | 5 | ELISA | 1 | 4 | 20.0 |
|  |  |  | 2025 | 1 | ELISA | 0 | 1 | 0 |
|  | Khmelnytska Oblast | NNP «Podilski Tovtry» | 2024 | 1 | ELISA | 0 | 1 | 0 |
|  | Poltava Oblast | RLP Nizhnevorsklyanskiy | 2023 | 10 | ELISA | 0 | 10 | 0 |
|  |  |  | 2024 | 21 | ELISA | 0 | 21 | 0 |
|  |  |  | 2025 | 3 | ELISA | 0 | 3 | 0 |
|  | Odesa Oblast | NPP Tuzlivski Lymany | 2024 | 1 | ELISA | 0 | 1 | 0 |
| Spotted Flycatcher *Muscicapa striata* | Poltava Oblast | RLP Nizhnevorsklyanskiy | 2023 | 2 | ELISA | 0 | 2 | 0 |
|  |  |  | 2024 | 1 | ELISA | 0 | 1 | 0 |
| Stonechat *Saxicola torquata* | Poltava Oblast | NPP Tuzlivski Lymany | 2024 | 2 | ELISA | 0 | 2 | 0 |
| Thrush Nightingale *Luscinia luscinia* | Poltava Oblast | RLP Nizhnevorsklyanskiy | 2023 | 2 | ELISA | 0 | 2 | 0 |
|  | Odesa Oblast | Trapivka-2 | 2024 | 1 | ELISA | 0 | 1 | 0 |
| **Oriolidae** | | | | | | | | |
| Golden Oriole *Oriolus oriolus* | Poltava Oblast | RLP Nizhnevorsklyanskiy | 2023 | 4 | ELISA | 0 | 4 | 0 |
| **Panuridae** | | | | | | | | |
| Bearded Tit *Panurus biarmicus* | Odesa Oblast | NPP Tuzlivski Lymany | 2024 | 2 | ELISA | 0 | 2 | 0 |
| **Paridae** | | | | | | | | |
| Blue Tit *Parus caeruleus* | Poltava Oblast | RLP Nizhnevorsklyanskiy | 2025 | 1 | ELISA | 0 | 1 | 0 |
| Great Tit *Parus major* | Kharkiv Oblast | Pershotravneve | 2023 | 11 | ELISA | 0 | 11 | 0 |
|  |  |  | 2024 | 15 | ELISA | 0 | 15 | 0 |
|  |  |  | 2025 | 8 | ELISA | 0 | 8 | 0 |
|  | Poltava Oblast | RLP Nizhnevorsklyanskiy | 2023 | 7 | ELISA | 0 | 7 | 0 |
|  |  |  | 2024 | 1 | ELISA | 0 | 1 | 0 |
|  | Kyiv Oblast | Balyko-Schynsenka | 2023 | 8 | ELISA | 0 | 8 | 0 |
|  | Odesa Oblast | Lyman | 2024 | 1 | ELISA | 0 | 1 | 0 |
|  |  | Trapivka | 2024 | 1 | ELISA | 0 | 1 | 0 |
|  |  | Trapivka-2 | 2024 | 2 | ELISA | 0 | 2 | 0 |
| **Passeridae** | | | | | | | | |
| House Sparrow *Passer domesticus* | Kharkiv Oblast | Poultry farm №1 | 2004 | 2 | Н1-Н14 | 0 | 2 | - |
|  |  | Poultry farm №2 | 2004 | 12 | Н1-Н14 | 0 | 12 | - |
|  |  | Poultry farm №4 | 2004 | 10 | Н1-Н14 | 0 | 10 | - |
|  |  | Pershotravneve | 2023 | 8 | ELISA | 0 | 8 | 0 |
|  |  |  | 2024 | 1 | ELISA | 0 | 1 | 0 |
|  |  |  | 2025 | 1 | ELISA | 0 | 1 | 0 |
| Spanish Sparrow *Passer hispaniolensis* | Odesa Oblast | Trapivka | 2024 | 3 | ELISA | 0 | 3 | 0 |
|  |  | Trapivka-2 | 2024 | 3 | ELISA | 0 | 3 | 0 |
| Tree Sparrow *Passer montanus* | Poltava Oblast | RLP Nizhnevorsklyanskiy | 2023 | 14 | ELISA | 0 | 14 | 0 |
|  |  |  | 2024 | 2 | ELISA | 0 | 2 | 0 |
|  |  |  | 2025 | 7 | ELISA | 0 | 7 | 0 |
|  | Odesa Oblast | Lyman | 2024 | 1 | ELISA |  |  |  |
|  |  | Trapivka-2 | 2024 | 5 | ELISA |  |  |  |
| **Sittidae** | | | | | | | | |
| Nuthatch *Sitta europaea* | Kharkiv Oblast | Pershotravneve | 2025 | 1 | ELISA | 0 | 1 | 0 |
| **Sturnidae** | | | | | | | | |
| Starling *Sturnus vulgaris* | Zaporizhzhia Region, | Davydivka | 2007 | 1 | Н1-Н14 | 0 | 1 | - |
|  | Poltava Oblast | RLP Nizhnevorsklyanskiy | 2023 | 1 | ELISA | 0 | 1 | 0 |
|  | Odesa Oblast | NPP Tuzlivski Lymany | 2024 | 2 | ELISA | 0 | 2 | 0 |
|  |  | Tarutynskyy step | 2024 | 1 | ELISA | 0 | 1 | 0 |
| **Sylviidae** | | | | | | | | |
| Great Reed Warbler *Acrocephalus arundinaceus* | Zaporizhzhia Region | Davydivka | 2007 | 5 | Н1-Н14 | 0 | 5 | - |
|  | Poltava Oblast | RLP Nizhnevorsklyanskiy | 2023 | 4 | ELISA | 0 | 4 | 0 |
|  | Odesa Oblast | Lyman | 2024 | 3 | ELISA | 0 | 3 | 0 |
|  |  | Trapivka | 2024 | 1 | ELISA | 0 | 1 | 0 |
|  |  | Trapivka-2 | 2024 | 1 | ELISA | 0 | 1 | 0 |
| Barred Warbler *Sylvia nisoria* | Poltava Oblast | RLP Nizhnevorsklyanskiy | 2023 | 2 | ELISA | 0 | 2 | 0 |
| Blackcap *Sylvia atricapilla* | Odesa Oblast | Trapivka | 2024 | 1 | ELISA | 0 | 1 | 0 |
|  |  | Lyman | 2024 | 1 | ELISA | 0 | 1 | 0 |
|  |  | Trapivka-2 | 2024 | 3 | ELISA | 0 | 3 | 0 |
|  | Poltava Oblast | RLP Nizhnevorsklyanskiy | 2024 | 2 | ELISA | 0 | 2 | 0 |
|  | Khmelnytska Oblast | NNP «Podilski Tovtry» | 2024 | 1 | ELISA | 1 | 0 | 100 |
| Chiffchaff *Phylloscopus collybita* | Odesa Oblast | NPP Tuzlivski Lymany | 2024 | 1 | ELISA | 0 | 1 | 0 |
| Marsh Warbler *Acrocephalus palustris* | Odesa Oblast | Lyman | 2024 | 1 | ELISA | 0 | 1 | 0 |
| Paddyfield Warbler *Acrocephalus agricola* | Odesa Oblast | Lyman | 2024 | 2 | ELISA | 0 | 2 | 0 |
| Reed Warbler *Acrocephalus scirpaceus* | Odesa Oblast | Lyman | 2024 | 2 | ELISA | 0 | 2 | 0 |
|  |  | Trapivka | 2024 | 1 | ELISA | 0 | 1 | 0 |
| River Warbler *Locustella fluviatilis* | Odesa Oblast | NPP Tuzlivski Lymany | 2024 | 1 | ELISA | 0 | 1 | 0 |
| Sedge Warbler *Acrocephalus schoenobaenus* | Odesa Oblast | NPP Tuzlivski Lymany | 2024 | 1 | ELISA | 0 | 1 | 0 |
|  |  | Lyman | 2024 | 1 | ELISA | 0 | 1 | 0 |
| Whitethroat *Sylvia communis* | Poltava Oblast | RLP Nizhnevorsklyanskiy | 2023 | 2 | ELISA | 0 | 2 | 0 |
|  | Odesa Oblast | Trapivka | 2024 | 3 | ELISA | 0 | 3 | 0 |
|  |  | Lyiman | 2024 | 2 | ELISA | 0 | 2 | 0 |
|  |  | Trapivka-2 | 2024 | 1 | ELISA | 0 | 1 | 0 |
| **Eggs yolk** | | | | | | | | |
| **Corvidae** | | | | | | | | |
| Jay *Garrulus glandarius* | Kharkiv region | Kharkiv (Hidropark) | 2006 | 1 | Н1 | 1 | 0 | 100,0 |
|  |  |  |  |  | Н8 | 1 | 0 | 100,0 |
|  | Kirovograd Oblast | Ukrainka | 2025 | 1 | ELISA | 0 | 1 | 0 |
| Rook *Corvus frugilegus* | Odesa region | Odesa | 2005 | 8 | Н1-Н14 | 0 | 8 | - |
| Hooded Crow *Corvus cornix* | Kherson region | NNP «Dzharylhatskyi» | 2006 | 3 | Н1-Н14 | 0 | 3 | - |
| Magpie *Pica pica* | Kharkiv region | Kharkiv (Saltivka) | 2006 | 2 | Н1-Н14 | 0 | 2 | - |
|  |  | Haidary | 2006 | 2 | Н1-Н14 | 0 | 2 | - |
|  |  | Hineievka | 2006 | 5 | Н1-Н14 | 0 | 5 | - |
|  |  | Vysokyi | 2006 | 1 | Н1-Н14 | 0 | 1 | - |
|  | Kherson region | NNP «Dzharylhatskyi» | 2006 | 3 | Н1-Н14 | 0 | 3 | - |
| **Emberizidae** | | | | | | | | |
| Yellowhammer *Emberiza citrinella* | Kirovograd Oblast | Ukrainka | 2025 | 1 | ELISA | 0 | 1 | 0 |
| **Fringillidae** | | | | | | | | |
| Chaffinch *Fringilla coelebs* | Kharkiv region | Hineievka | 2006 | 3 | Н1-Н14 | 0 | 3 | - |
|  | Khmelnytsk Oblast | Maliivtsi | 2023 | 1 | ELISA | 0 | 1 | 0 |
| Greenfinch *Chloris chloris* | Kharkiv region | Haidary | 2006 | 1 | Н1-Н14 | 0 | 1 | - |
|  | Sumy region | NNP «Hetmanskyi» | 2006 | 1 | Н1-Н14 | 0 | 1 | - |
|  | Poltava Oblast | RLP Nizhnevorsklyanskiy | 2023 | 3 | ELISA | 0 | 3 | 0 |
| Hawfinch *Coccothraustes coccothraustes* | Kharkiv region | Haidary | 2006 | 3 | Н1-Н14 | 0 | 3 | - |
| **Hirundinidae** | | | | | | | | |
| Swallow *Hirundo rustica* | Kharkiv region | Poultry farm №1 | 2004 | 6 | Н1-Н14 | 0 | 6 | - |
|  |  | Poultry farm №3 | 2004 | 10 | Н1-Н14 | 0 | 10 | - |
| **Laniidae** | | | | | | | | |
| Red-backed Shrike *Lanius collurio* | Kharkiv region | Haidary | 2006 | 3 | Н1-Н14 | 0 | 3 | - |
|  |  | Zanky | 2006 | 1 | Н1-Н14 | 0 | 1 | - |
|  |  | Hineievka | 2006 | 1 | Н1-Н14 | 0 | 1 | - |
| **Muscicapidae** | | | | | | | | |
| Blackbird *Turdus merula* | Kharkiv region | Haidary | 2006 | 4 | Н1-Н14 | 0 | 4 | - |
|  |  | Hineievka | 2006 | 2 | Н1 | 1 | 1 | 50,0 |
|  | Khmelnytska Oblast | Maliivtsi | 2023 | 1 | ELISA | 0 | 1 | 0 |
|  | Kharkiv Oblast | Gaidary | 2023 | 1 | ELISA | 0 | 1 | 0 |
|  | Kirovograd Oblast | Ukrainka | 2025 | 1 | ELISA | 0 | 1 | 0 |
| Collared Flycatcher *Ficedula albicollis* | Sumy region | Vakalivshchyna, | 2004 | 3 | Н1-Н14 | 0 | 3 | - |
|  |  | NNP «Hetmanskyi» | 2006 | 9 | Н1 | 2 | 7 | 22,2 |
|  | Kharkiv region | Haidary | 2006 | 11 | Н1-Н14 | 0 | 11 | - |
|  |  | RLP «Feldman ecopark» | 2006 | 9 | Н1-Н14 | 0 | 9 | - |
|  |  | NNP «Homilshanski lisy» | 2006 | 6 | Н1-Н14 | 0 | 6 | - |
|  |  | Hineievka | 2006 | 2 | Н1-Н14 | 0 | 2 | - |
| Song Thrush *Turdus philomelos* | Poltava Oblast | RLP Nizhnevorsklyanskiy | 2023 | 1 | ELISA | 0 | 1 | 0 |
|  | Khmelnytska Oblast | Maliivtsi | 2023 | 1 | ELISA | 0 | 1 | 0 |
|  | Sumy region | NNP «Hetmanskyi» | 2006 | 2 | Н1 | 2 | 0 | 100,0 |
|  |  | Vakalivshchyna | 2006 | 1 | Н1-Н14 | 0 | 1 | - |
|  | Kharkiv region | Haidary | 2006 | 11 | Н1-Н14 | 0 | 11 | - |
|  |  | Kharkiv (Hydropark) | 2006 | 1 | Н1-Н14 | 0 | 1 | - |
|  |  | NNP «Homilshanski lisy» | 2006 | 4 | Н1 | 2 | 2 | 50,0 |
|  |  |  |  |  | Н2 | 1 | 3 | 25,0 |
|  |  |  |  |  | Н8 | 1 | 3 | 25,0 |
|  |  |  | 2007 | 1 | Н1-Н14 | 0 | 1 | - |
| Black Redstart *Phoenicurus ochruros* | Kharkiv region | Haidary | 2006 | 1 | Н1-Н14 | 0 | 1 | - |
| Robin *Erithacus rubecula* | Kharkiv region | Haidary | 2006 | 1 | Н1-Н14 | 0 | 1 | - |
|  | Sumy region | NNP «Hetmanskyi» | 2006 | 9 | Н1-Н14 | 0 | 9 | - |
| Fieldfare *Turdus pilaris* | Kharkiv region | Kharkiv (Hydropark) | 2006 | 6 | Н1-Н14 | 0 | 6 | - |
|  |  | Hineievka | 2006 | 3 | Н1-Н14 | 0 | 3 | - |
| Bluethroat *Luscinia svecica* | Kharkiv region | Haidary | 2007 | 1 | Н5 | 1 | 0 | 100,0 |
|  |  |  |  |  | Н14 | 1 | 0 | 100,0 |
| Pied Flycatcher *Ficedula hypoleuca* | Kharkiv region | Haidary | 2007 | 4 | Н1-Н14 | 0 | 4 | - |
|  |  | NNP «Homilshanski lisy» | 2007 | 4 | Н1-Н14 | 0 | 4 | - |
| **Paridae** | | | | | | | | |
| Great Tit *Parus major* | Poltava Oblsdt | Horishni Plavni | 2023 | 1 | ELISA | 0 | 1 | 0 |
|  | Kharkiv region | RLP «Feldman ecopark» | 2006 | 1 | Н1-Н14 | 0 | 1 | - |
|  |  | NNP «Homilshanski lisy» | 2006 | 14 | Н1-Н14 | 0 | 14 | - |
|  | Sumy region | NNP «Hetmanskyi» | 2006 | 7 | Н1-Н14 | 0 | 7 | - |
|  |  | Vakalivshchyna | 2006 | 2 | Н1 | 1 | 1 | 50,0 |
|  |  |  |  |  | Н2 | 1 | 1 | 50,0 |
|  |  |  |  |  | Н8 | 1 | 1 | 50,0 |
| Blue Tit *Parus caeruleus* | Kharkiv region | RLP «Feldman ecopark» | 2006 | 2 | Н1-Н14 | 0 | 2 | - |
| Marsh Tit *Parus palustris* | Kharkiv region | NNP «Homilshanski lisy» | 2006 | 3 | Н1-Н14 | 0 | 3 | - |
| **Passeridae** | | | | | | | | |
| House Sparrow *Passer domesticus* | Kharkiv region | Poultry farm №1 | 2004 | 40 | Н1 | 6 | 34 | 15,0 |
|  |  |  |  | 40 | Н8 | 5 | 35 | 12,5 |
|  |  | Poultry farm №3 | 2004 | 29 | Н1-Н14 | 0 | 29 | - |
| **Sylviidae** | | | | | | | | |
| Blackcap *Sylvia atricapilla* | Sumy region | Vakalivshchyna | 2006 | 2 | Н1-Н14 | 0 | 2 | - |
|  | Kirovograd Oblast | Ukrainka | 2025 | 1 | ELISA | 0 | 1 | 0 |
