# Supplementary material for: Wild passerines as potential carriers and sources of avian influenza viruses in Ukraine
Source: Front Microbiol. 2026 Jan 20;16:1736454. doi: 10.3389/fmicb.2025.1736454 (PMC12864440; doi:10.3389/fmicb.2025.1736454)
Supplement: Supplementary file 3 [file Table_3.docx]

**Supplementary table**

Table S3. Results of PCR prevalence to AIV in Passerines in Ukraine in 2004-2025 (full data including positive and negative results).

| Bird species | Region | Location | Year | Samples, total | PCR | | Prevalence, % |
| --- | --- | --- | --- | --- | --- | --- | --- |
|  |  |  |  |  | Pos | Neg |  |
| **Aegithalidae** | | | | | | | |
| Long-tailed Tit *Aegithalos caudatus* | Poltava Oblast | RLP «Nyzhnovorsklianskyi» | 2024 | 2 | 0 | 2 | 0 |
|  |  |  | 2025 | 3 | 0 | 3 | 0 |
|  | Lviv Oblast | NPP Yavorivskyi | 2024 | 11 | 0 | 11 | 0 |
| **Bombycillidae** | | | | | | | |
| Waxwing *Bombycilla garrulus* | Kharkiv Oblast | Pershotravneve | 2023 | 10 | 0 | 10 | 0 |
| **Certhiidae** | | | | | | | |
| Treecreeper *Certhia familiaris* | Kyiv Oblast | Hlyboki Balyky | 2023 | 1 | 0 | 1 | 0 |
| **Corvidae** | | | | | | | |
| Jay *Garrulus glandarius* | Poltava Oblast | RLP Nyzhnovorsklianskyi | 2023 | 1 | 0 | 1 | 0 |
|  |  |  | 2024 | 2 | 0 | 2 | 0 |
|  |  |  | 2025 | 4 | 0 | 4 | 0 |
|  | Kyiv Oblast | Hlyboki Balyky | 2023 | 2 | 0 | 2 | 0 |
|  | Kharkiv Oblast | Pershotravneve | 2024 | 1 | 0 | 1 | 0 |
|  | Lviv Oblast | NPP Yavorivskyi | 2024 | 1 | 0 | 1 | 0 |
| **Emberizidae** | | | | | | | |
| Yellowhammer *Emberiza citrinella* | Poltava Oblast | RLP Nyzhnovorsklianskyi | 2023 | 3 | 0 | 3 | 0 |
|  |  |  | 2024 | 6 | 0 | 6 | 0 |
|  |  |  | 2025 | 2 | 0 | 2 | 0 |
| **Fringillidae** | | | | | | | |
| Chaffinch *Fringilla coelebs* | Kharkiv Oblast | Gaidary | 2023 | 22 | 0 | 22 | 0 |
|  |  | Pershotravneve | 2024 | 5 | 1 | 4 | 20 |
|  | Khmelnytska Oblast | Maliivtsi | 2023 | 5 | 0 | 5 | 0 |
|  | Poltava Oblast | RLP Nyzhnovorsklianskyi | 2024 | 17 | 0 | 17 | 0 |
|  |  |  | 2025 | 8 | 2 | 6 | 25 |
| Goldfinch *Carduelis carduelis* | Kharkiv Oblast | Gaidary | 2023 | 6 | 1 | 5 | 16,6 |
|  | Poltava Oblast | RLP Nyzhnovorsklianskyi | 2023 | 6 | 0 | 6 | 0 |
|  |  |  | 2024 | 2 | 0 | 2 | 0 |
|  |  |  | 2025 | 1 | 0 | 1 | 0 |
| Greenfinch *Chloris chloris* | Kharkiv Oblast | Gaidary | 2023 | 8 | 0 | 8 | 0 |
|  | Khmelnytska Oblast | Maliivtsi | 2023 | 2 | 0 | 2 | 0 |
|  | Poltava Oblast | RLP Nyzhnovorsklianskyi | 2023 | 43 | 2 | 41 | 4,6 |
|  |  |  | 2024 | 21 | 0 | 21 | 0 |
|  |  |  | 2025 | 30 | 3 | 27 | 10 |
| Hawfinch *Coccothraustes coccothraustes* | Kharkiv Oblast | Gaidary | 2023 | 11 | 0 | 11 | 0 |
|  |  | Pershotravneve | 2024 | 1 | 0 | 1 | 0 |
|  | Khmelnytska Oblast | Maliivtsi | 2023 | 2 | 0 | 2 | 0 |
|  | Poltava Oblast | RLP Nyzhnovorsklianskyi | 2023 | 3 | 0 | 3 | 0 |
|  |  |  | 2024 | 3 | 0 | 3 | 0 |
|  |  |  | 2025 | 7 | 0 | 7 | 0 |
|  | Kirovohradska Oblast | Ukrainka | 2025 | 1 | 0 | 1 | 0 |
| **Hirundinidae** | | | | | | | |
| House Martin *Delichon urbica* | Poltava Oblast | RLP Nyzhnovorsklianskyi | 2023 | 15 | 0 | 15 | 0 |
| Swallow *Hirundo rustica* | Poltava Oblast | RLP Nyzhnovorsklianskyi | 2023 | 18 | 0 | 18 | 0 |
| Laniidae | | | | | | | |
| Lesser Grey Shrike *Lanius minor* | Poltava Oblast | Luchky, RLP «Nyzhnovorsklianskyi» | 2024 | 1 | 0 | 1 | 0 |
| Red-backed Shrike *Lanius collurio* | Poltava Oblast | Luchky, RLP «Nyzhnovorsklianskyi» | 2023 | 9 | 0 | 9 | 0 |
| **Motacillidae** | | | | | | | |
| Pied Wagtail *Motacilla alba* | Poltava Oblast | RLP Nyzhnovorsklianskyi | 2023 | 1 | 0 | 1 | 0 |
|  |  |  | 2024 | 1 | 0 | 1 | 0 |
|  |  |  | 2025 | 1 | 0 | 1 | 0 |
| Tree Pipit *Anthus trivialis* | Kirovohradska Oblast | Ukrainka | 2025 | 1 | 0 | 1 | 0 |
| Yellow Wagtail *Motacilla flava* | Odesa Oblast | Trapivka | 2024 | 1 | 0 | 1 | 0 |
| **Muscicapidae** | | | | | | | |
| Black Redstart *Phoenicurus ochruros* | Kharkiv Oblast | Gaidary | 2023 | 1 | 0 | 1 | 0 |
|  | Lviv Oblast | NPP Yavorivskyi | 2024 | 1 | 0 | 1 | 0 |
| Blackbird *Turdus merula* | Kharkiv Oblast | Gaidary | 2023 | 11 | 2 | 9 | 18,1 |
|  | Kharkiv Oblast | Pershotravneve | 2024 | 9 | 1 | 8 | 11,1 |
|  | Khmelnytska Oblast | Maliivtsi | 2023 | 6 | 0 | 6 | 0 |
|  | Poltava Oblast | RLP Nyzhnovorsklianskyi | 2023 | 11 | 1 | 10 | 9 |
|  | Poltava Oblast |  | 2024 | 12 | 0 | 12 | 0 |
|  | Poltava Oblast |  | 2025 | 10 | 0 | 10 | 0 |
|  | Kyiv Oblast | Hlyboki Balyky | 2023 | 2 | 0 | 2 | 0 |
|  | Khmelnytska Oblast | NPP «Podilski  Tovtry» | 2024 | 1 | 0 | 1 | 0 |
|  | Kirovohradska Oblast | Ukrainka | 2025 | 3 | 0 | 3 | 0 |
| Bluethroat *Luscinia svecica* | Poltava Oblast | RLP Nyzhnovorsklianskyi | 2023 | 1 | 0 | 1 | 0 |
| Collared Flycatcher *Ficedula albicollis* | Khmelnytska Oblast | Maliivtsi | 2023 | 4 | 0 | 4 | 0 |
|  | Odesa Oblast | Lyman | 2024 | 5 | 0 | 5 | 0 |
|  |  | Trapivka-2 | 2024 | 2 | 1 | 1 | 50 |
| Pied Flycatcher *Ficedula hypoleuca* | Poltava Oblast | RLP Nyzhnovorsklianskyi | 2024 | 1 | 0 | 1 | 0 |
|  |  |  | 2025 | 1 | 0 | 1 | 0 |
|  | Kirovohradska Oblast | Ukrainka | 2025 | 1 | 0 | 1 | 0 |
| Robin *Erithacus rubecula* | Kharkiv Oblast | Gaidary | 2023 | 10 | 2 | 8 | 20 |
|  |  | Pershotravneve | 2024 | 4 | 0 | 4 | 0 |
|  | Poltava Oblast | RLP Nyzhnovorsklianskyi | 2023 | 1 | 0 | 1 | 0 |
|  |  |  | 2024 | 24 | 1 | 23 | 4,1 |
|  |  |  | 2025 | 17 | 0 | 17 | 0 |
|  | Kyiv Oblast | Hlyboki Balyky | 2023 | 9 | 0 | 9 | 0 |
|  | Khmelnytska Oblast | NPP «Podilski  Tovtry» | 2024 | 6 | 0 | 6 | 0 |
|  | Lviv Oblast | Yavorivskyi NPP | 2024 | 3 | 0 | 3 | 0 |
| Song Thrush *Turdus philomelos* | Kharkiv Oblast | Gaidary | 2023 | 17 | 0 | 17 | 0 |
|  |  | Pershotravneve | 2024 | 7 | 0 | 7 | 0 |
|  | Poltava Oblast | RLP Nyzhnovorsklianskyi | 2023 | 13 | 0 | 13 | 0 |
|  |  |  | 2024 | 21 | 0 | 21 | 0 |
|  |  |  | 2025 | 7 | 1 | 6 | 14,2 |
|  | Khmelnytska Oblast | NPP «Podilski  Tovtry» | 2024 | 1 | 0 | 1 | 0 |
| Spotted Flycatcher *Muscicapa striata* | Poltava Oblast | RLP Nyzhnovorsklianskyi | 2023 | 3 | 0 | 3 | 0 |
|  |  |  | 2024 | 1 | 0 | 1 | 0 |
| Stonechat *Saxicola torquata* | Poltava Oblast | RLP Nyzhnovorsklianskyi | 2024 | 1 | 0 | 1 | 0 |
| Thrush Nightingale *Luscinia luscinia* | Poltava Oblast | RLP Nyzhnovorsklianskyi | 2023 | 2 | 0 | 2 | 0 |
|  |  |  | 2024 | 1 | 0 | 1 | 0 |
|  | Odesa Oblast | Liman | 2024 | 1 | 0 | 1 | 0 |
|  |  | Trapivka-2 | 2024 | 1 | 0 | 1 | 0 |
| Whinchat *Saxicola rubetra* | Poltava Oblast | RLP Nyzhnovorsklianskyi | 2023 | 1 | 0 | 1 | 0 |
| **Oriolidae** | | | | | | | |
| Golden Oriole *Oriolus oriolus* | Poltava Oblast | RLP Nyzhnovorsklianskyi | 2023 | 4 | 0 | 4 | 0 |
| **Panuridae** | | | | | | | |
| Bearded Tit *Panurus biarmicus* | Odesa Oblast | NPP Tuzlivski Lymany | 2024 | 2 | 2 | 0 | 100 |
| **Paridae** | | | | | | | |
|  | Kyiv Oblast | Hlyboki Balyky | 2023 | 5 | 0 | 5 | 0 |
| Blue Tit *Parus caeruleus* | Kharkiv Oblast | Gaidary | 2023 | 3 | 0 | 3 | 0 |
|  |  | Pershotravneve | 2023 | 5 | 0 | 5 | 0 |
|  |  |  | 2024 | 2 | 0 | 2 | 0 |
|  | Poltava Oblast | RLP Nyzhnovorsklianskyi | 2024 | 3 | 0 | 3 | 0 |
|  |  |  | 2025 | 4 | 0 | 4 | 0 |
|  | Khmelnytska Oblast | NPP «Podilski  Tovtry» | 2024 | 2 | 0 | 2 | 0 |
| Great Tit *Parus major* | Kharkiv Oblast | Dergachi | 2022 | 2 | 0 | 2 | 0 |
|  |  | Manchenki | 2022 | 55 | 0 | 55 | 0 |
|  |  | Pershotravneve | 2023 | 22 | 5 | 17 | 22,7 |
|  |  |  | 2024 | 14 | 0 | 14 | 0 |
|  |  | Gaidary | 2023 | 29 | 2 | 27 | 7 |
|  | Poltava Oblast | RLP Nyzhnovorsklianskyi | 2023 | 13 | 0 | 13 | 0 |
|  |  |  | 2024 | 16 | 1 | 15 | 6,25 |
|  |  |  | 2025 | 23 | 1 | 22 | 4,3 |
|  | Kyiv Oblast | Hlyboki Balyky | 2023 | 15 | 0 | 15 | 0 |
|  | Lviv Oblast | NPP Yavorivskyi | 2024 | 17 | 1 | 16 | 5,8 |
|  | Odesa Oblast | Liman | 2024 | 2 | 0 | 2 | 0 |
|  |  | Trapivka | 2024 | 1 | 0 | 1 | 0 |
|  |  | Trapivka-2 | 2024 | 5 | 1 | 4 | 20 |
|  | Khmelnytska Oblast | NPP «Podilski  Tovtry» | 2024 | 2 | 0 | 2 | 0 |
|  | Kirovohradska Oblast | Ukrainka | 2025 | 1 | 0 | 1 | 0 |
| Marsh Tit *Parus palustris* | Kharkiv Oblast | Gaidary | 2023 | 1 | 0 | 1 | 0 |
|  |  | Pershotravneve | 2024 | 3 | 0 | 3 | 0 |
|  | Kyiv Oblast | Hlyboki Balyky | 2023 | 3 | 0 | 3 | 0 |
|  | Khmelnytska Oblast | NPP «Podilski  Tovtry» | 2024 | 3 | 0 | 3 | 0 |
|  | Lviv Oblast | NPP Yavorivskyi | 2024 | 1 | 0 | 1 | 0 |
| **Passeridae** | | | | | | | |
| House Sparrow *Passer domesticus* | Kharkiv Oblast | Dergachi | 2022 | 33 | 0 | 33 | 0 |
|  |  | Manchenki | 2022 | 29 | 0 | 29 | 0 |
|  |  | Pershotravneve | 2023 | 8 | 1 | 7 | 12,5 |
| Tree Sparrow *Passer montanus* | Poltava Oblast | RLP Nyzhnovorsklianskyi | 2023 | 29 | 0 | 29 | 0 |
|  |  |  | 2024 | 12 | 0 | 12 | 0 |
|  |  |  | 2025 | 24 | 0 | 24 | 0 |
|  | Odesa Oblast | Liman | 2024 | 1 | 0 | 1 | 0 |
|  |  | Trapivka-2 | 2024 | 3 | 0 | 3 | 0 |
| **Regulidae** | | | | | | | |
| Goldcrest *Regulus regulus* | Lviv Oblast | Yavorivskyi NPP | 2024 | 12 | 0 | 12 | 0 |
| **Sittidae** | | | | | | | |
| Nuthatch *Sitta europaea* | Khmelnytska Oblast | Maliivtsi | 2023 | 1 | 0 | 1 | 0 |
|  | Kyiv Oblast | Hlyboki Balyky | 2023 | 1 | 0 | 1 | 0 |
|  | Lviv Oblast | NPP Yavorivskyi | 2024 | 2 | 0 | 2 | 0 |
| **Sturnidae** | | | | | | | |
| Starling *Sturnus vulgaris* | Kharkiv Oblast | Dergachi | 2022 | 47 | 0 | 47 | 0 |
|  | Poltava Oblast | RLP Nyzhnovorsklianskyi | 2023 | 1 | 0 | 1 | 0 |
| **Sylviidae** | | | | | | | |
| Barred Warbler *Sylvia nisoria* | Poltava Oblast | RLP Nyzhnovorsklianskyi | 2023 | 4 | 0 | 4 | 0 |
| Blackcap *Sylvia atricapilla* | Khmelnytska Oblast | Maliivtsi | 2023 | 2 | 0 | 2 | 0 |
|  |  | NPP «Podilski  Tovtry» | 2024 | 4 | 0 | 4 | 0 |
|  | Poltava Oblast | RLP Nyzhnovorsklianskyi | 2023 | 6 | 0 | 6 | 0 |
|  |  |  | 2024 | 4 | 0 | 4 | 0 |
|  |  |  | 2025 | 8 | 1 | 7 | 12,5 |
|  | Odesa Oblast | Lyman | 2024 | 1 | 0 | 1 | 0 |
|  |  | Trapivka-2 | 2024 | 3 | 0 | 3 | 0 |
|  | Kirovohradska Oblast | Ukrainka | 2025 | 2 | 0 | 2 | 0 |
| Chiffchaff *Phylloscopus collybita* | Poltava Oblast | RLP Nyzhnovorsklianskyi | 2023 | 2 | 0 | 2 | 0 |
|  |  |  | 2024 | 4 | 0 | 4 | 0 |
|  |  |  | 2025 | 10 | 0 | 10 | 0 |
|  | Kharkiv Oblast | Pershotravneve | 2024 | 1 | 0 | 1 | 0 |
|  | Odesa Oblast | NPP Tuzlivski Lymany | 2024 | 1 | 1 | 0 | 100 |
|  | Kirovohradska Oblast | Ukrainka | 2025 | 1 | 0 | 1 | 0 |
| Great Reed Warbler *Acrocephalus arundinaceus* | Poltava Oblast | RLP Nyzhnovorsklianskyi | 2023 | 4 | 0 | 4 | 0 |
|  | Odesa Oblast | Liman | 2024 | 2 | 0 | 2 | 0 |
|  |  | Trapivka | 2024 | 1 | 0 | 1 | 0 |
|  |  | Trapivka-2 | 2024 | 1 | 0 | 1 | 0 |
| Icterine Warbler *Hippolais icterina* | Odesa Oblast | Trapivka-2 | 2024 | 1 | 0 | 1 | 0 |
| Lesser Whitethroat *Sylvia curruca* | Odesa Oblast | Liman | 2024 | 9 | 1 | 8 | 11,1 |
|  |  | Trapivka | 2024 | 11 | 0 | 11 | 0 |
|  |  | Trapivka-2 | 2024 | 4 | 1 | 3 | 25 |
|  | Poltava Oblast | RLP Nyzhnovorsklianskyi | 2025 | 1 | 1 | 0 | 100 |
| Marsh Warbler  *Acrocephalus palustris* | Odesa Oblast | Lyman | 2024 | 1 | 0 | 1 | 0 |
| Paddyfield Warbler *Acrocephalus agricola* | Odesa Oblast | Lyman | 2024 | 6 | 0 | 6 | 0 |
| Reed Warbler *Acrocephalus scirpaceus* | Odesa Oblast | Liman | 2024 | 16 | 1 | 15 | 6,25 |
|  |  | Trapivka | 2024 | 2 | 0 | 2 | 0 |
|  |  | Trapivka-2 | 2024 | 1 | 0 | 1 | 0 |
| Savi`s Warbler *Locustella luscinioides* | Odesa Oblast | Liman | 2024 | 1 | 1 | 0 | 100 |
| Sedge Warbler *Acrocephalus schoenobaenus* | Odesa Oblast | NPP Tuzlivski Lymany | 2024 | 2 | 2 | 0 | 100 |
| Whitethroat *Sylvia communis* | Poltava Oblast | RLP Nyzhnovorsklianskyi | 2023 | 9 | 0 | 9 | 0 |
|  | Odesa Oblast | Trapivka | 2024 | 3 | 0 | 3 | 0 |
| Willow Warbler *Phylloscopus trochilus* | Odesa Oblast | Liman | 2024 | 4 | 0 | 4 | 0 |
|  |  | Trapivka | 2024 | 3 | 0 | 3 | 0 |
|  |  | Trapivka-2 | 2024 | 1 | 0 | 1 | 0 |
| **Troglodytidae** | | | | | | | |
| Wren *Troglodytes troglodytes* | Poltava Oblast | RLP Nyzhnovorsklianskyi | 2024 | 1 | 0 | 1 | 0 |
|  | Lviv Oblast | Yavorivskyi NPP | 2024 | 2 | 0 | 2 | 0 |
